# Novel surveillance design exploiting network analysis for efficient, rapid detection of emerging *Clostridioides difficile* strains

**DOI:** 10.1038/s41467-026-72311-0

**Published:** 2026-08-26

**Authors:** Diane Pople, Tjibbe Donker, Olisaeloka Nsonwu, Dakshika Jeyaratnam, Dimple Chudasama, Russell Hope, Susan Hopkins, Mark H. Wilcox, David W. Eyre, A. Sarah Walker, Julie V. Robotham

**Affiliations:** 1 United Kingdom Health Security Agency, London, UK; 2 Medical Center–University of Freiburg, Institute for Infection Prevention and Control, Freiburg, Germany; 3 Guy’s and St Thomas’ NHS Foundation Trust, London, UK; 4 NIHR Health Protection Research Unit in Antimicrobial Resistance and Healthcare Associated Infections, University of Oxford, Oxford, UK; 5 Leeds Institute of Medical Research, University of Leeds, Leeds, UK; 6 Department of Microbiology, Leeds Teaching Hospitals NHS Trust, Leeds, UK; 7 Big Data Institute, Nuffield Department of Medicine, University of Oxford, Oxford, UK; 8 Oxford University Hospitals NHS Foundation Trust, Oxford, UK; 9 Modernising Medical Microbiology, Nuffield Department of Medicine, University of Oxford, Oxford, UK; 10 NIHR Biomedical Research Centre, University of Oxford, Oxford, UK

**Keywords:** Epidemiology, Clostridium difficile, Disease prevention

## Abstract

Whole genome sequencing is being introduced in England to support national surveillance of key hospital-acquired pathogens, starting with *Clostridioides difficile*, to facilitate novel strain identification and enable timely interventions. However sequencing capacity is limited. Symptomatic and asymptomatic patients attending multiple hospitals can act as inter-facility transmission vectors; here we therefore identify the empirical network of shared patients from analysing national admission data and simulate spread of a hypothetical novel strain. Algorithmically optimising detection, incorporating logistical constraints, we identify sentinel sites which detect a novel strain 27% faster than random sentinel selection, whilst sequencing <15% cases. Sensitivity and scenario analyses using a range of plausible pathogen characteristics and historical networks confirm epidemiologically- and longitudinally-robust sentinel selection and performance. The new surveillance system, established from our findings, benefits from stress-tested sentinel set selection to deliver rapid, efficient identification of novel strains within real world constraints, to inform control interventions, and provides a roadmap for future hospital-acquired pathogen surveillance.

## Abstract (web summary)

Using network analysis for inter-hospital spread and algorithmically optimising sentinel site selection, the authors design a surveillance system to rapidly detect emerging nosocomial pathogen strains within limited whole genome sequencing capacity.

## Introduction

Hospital-acquired infections place a significant burden on healthcare resources, accounting for an estimated 21% of bed-days in English hospitals in 2016/17^1^, a burden that can be exacerbated by increases in incidence of pathogen subtypes with hyper-virulence or resistance to existing control measures or treatment^2–4^, threatening effective healthcare provision. Surveillance goals, therefore, include monitoring incidence and subtypes to detect emergence or growth of variants of concern. The increasingly widespread adoption of whole genome sequencing (WGS) makes high precision detection and tracking of specific lineages possible, potentially facilitating implementation of more timely control measures. However, WGS is also costly, and capacity is limited. Hence, sequencing all or most cases of hospital-acquired infections of greatest clinical and operational concern is unlikely to be practically possible or optimal. Exploiting the likely dynamics of emergent subtypes across national hospital ecosystems could benefit the design of surveillance systems migrating to include WGS, optimising sentinel surveillance to maximise early emergent threat detection, given the limited sequencing capacity logistically available.

A common feature of modern healthcare is that many patients attend more than one provider^5–7^. For example, within the English National Health Service (NHS), the main healthcare provider, 1 in 12 of all acute hospital admissions in 2014/15 were discharged from another facility within the previous year^8^. Several important nosocomial infections, including *Clostridioides difficile* and key antimicrobial resistance threats such as Carbapenemase-producing Enterobacterales (CPE) or methicillin-resistant *Staphylococcus aureus* (MRSA), can be carried harmlessly as part of the commensal flora, and only occasionally cause severe disease, meaning that asymptomatic, colonised individuals can be sources of transmission to other patients^9–13^. Hence, by attending multiple hospitals, they can unwittingly act as vectors, spreading hospital-acquired infections between facilities. As an exemplar, *C. difficile* infection (CDI) is the leading cause of infectious diarrhoea in hospitalised patients and is associated with significant mortality and healthcare resource burden^14,15^. Within healthcare facilities, *C. difficile* can be transmitted faecal-orally, including via contact with fomites contaminated with *C. difficile* spores shed from those with or without symptomatic disease^9,10^. The contribution of patients attending multiple hospitals to inter-hospital *C. difficile* transmission has been demonstrated for the toxigenic ribotype 027 (RT027) strain^16,17^ associated with increased incidence and mortality^18^, and is supported by correlations between CDI cases and inter-hospital transfers^19–22^.

The structure of connections between facilities formed by patient movements can therefore provide insight into inter-hospital pathogen dynamics, and can be interrogated using network analysis^5–7^. Previous studies seeking to improve efficiency in hospital-based surveillance systems through network analysis have considered local/global network measures^8,23^, hospital characteristic-based heuristics^24,25^, and algorithms applied to information from simulated outbreaks^24,25^. Here, we extended the latter approach, using simulations of a hypothetical novel *C. difficile* ‘strain X’ spreading between healthcare providers via patients as vectors for inter-facility transmission with an algorithm prioritising its prompt detection, to design a new surveillance system based on a limited number of sentinel sites supplying samples for WGS, extending the previous methodology to incorporate real-world logistics and implementational considerations. We used CDI as an exemplar because, after a period of stability, cases in England have risen to a 10-year high (driven by hospital cases)^26^ with no clear explanation^27^, highlighting the importance of greater active surveillance to detect novel strains. These insights into inter-hospital pathogen dynamics form the basis of the new genomic sentinel system introduced in England in 2025, directly designed to provide early warning of emergent strains causing CDI, but generalisable to other healthcare-associated infections and applicable to other countries.

We present below the development and evaluation of this algorithmically optimised sentinel set selection, used to inform the new CDI surveillance programme.

## Results

**Fig. 1:**
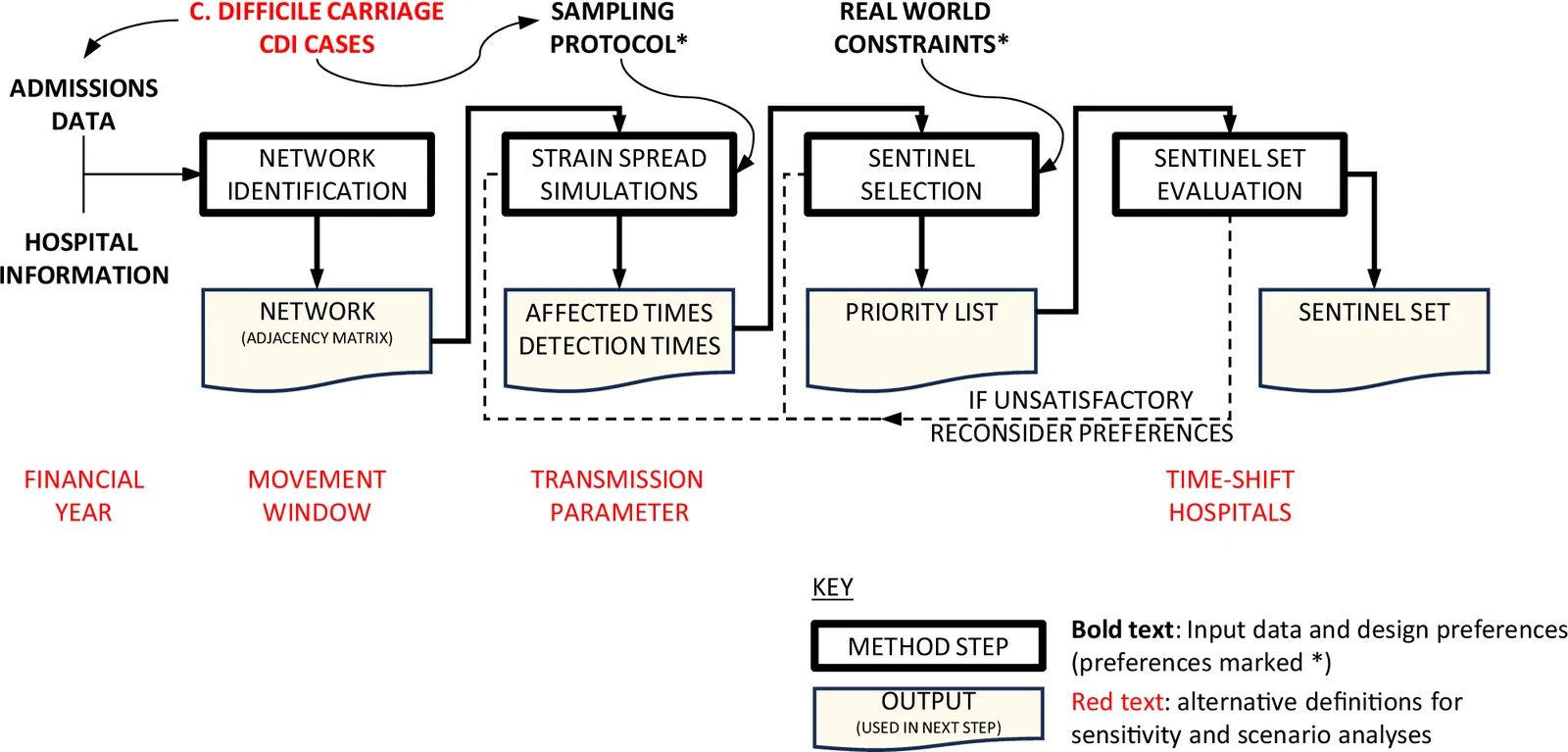
Outline of methodological approach.

Figure 1 summarises our overall approach to sentinel site selection, encompassing empirical network identification, simulation of a hypothetical novel strain’s spread across the network, sentinel site selection and evaluation, including multiple sensitivity and scenario analyses to stress-test the proposed surveillance system before implementation.

### Empirical network identification and characteristics

**Fig. 2:**
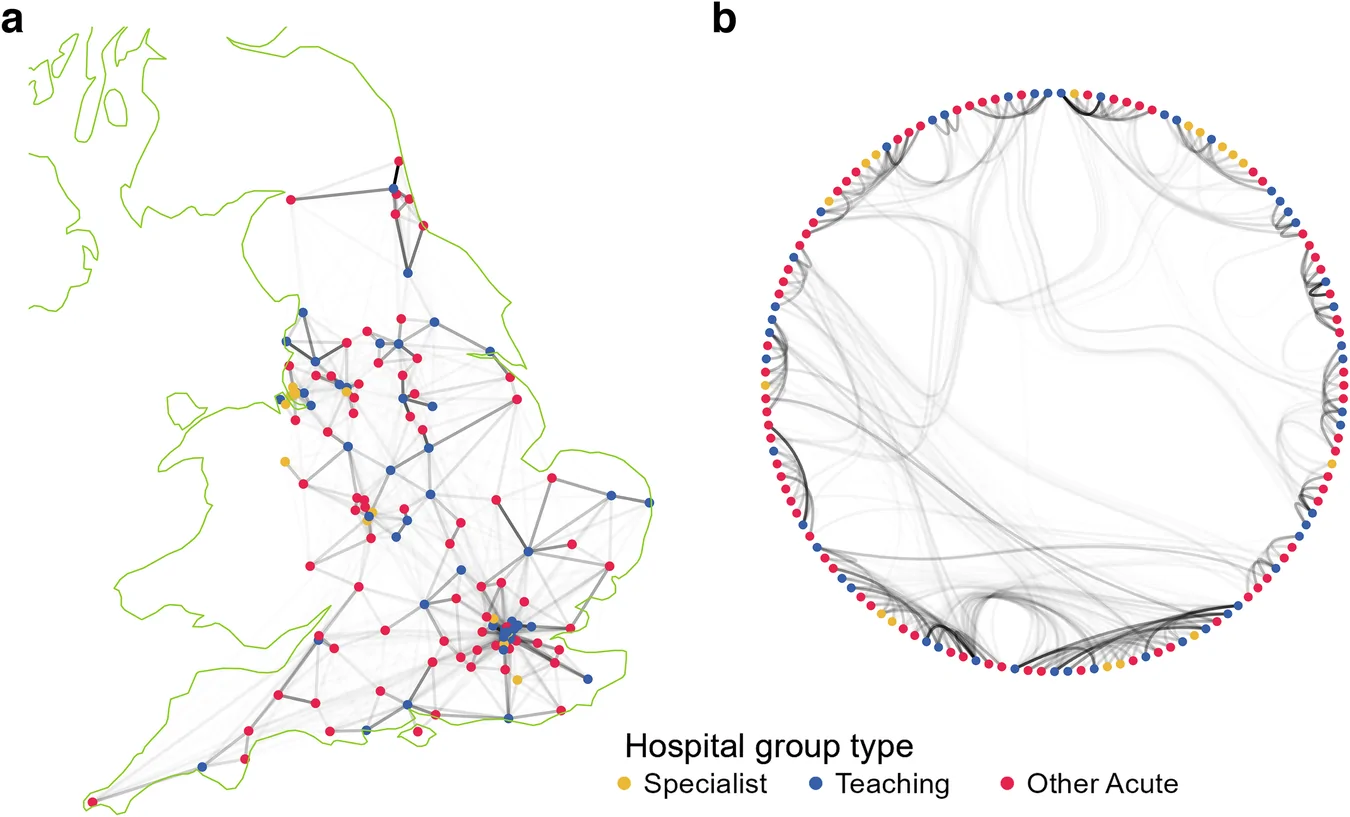
Network of patient movements for the financial year FY2022/23. The network of prior movements for admissions between 1 April 2022 and 31 March 2023 (window between inpatient hospital stays (*w*) = 182 days) depicted (**a**) geographically and (**b**) as a chord diagram. Note: darker lines have higher edge weight (i.e. numbers of shared inpatients). Showing only links with shared patient admission at least monthly (mean) in at least one direction.

We first identified a directed, weighted network of inter-hospital patient movements in England from empirical overnight inpatient admission data^28^ for the financial year 1 April 2022–31 March 2023 (FY2022/23) using the HospitalNetwork R package^29^. Network vertices (or nodes) were acute hospital trusts: secondary/tertiary care-provider organisations within NHS England who are responsible for the control and reporting of CDI cases^30,31^, subsequently denoted ‘hospital groups’. A directed network edge, *H*_*i*_→*H*_*j*_, was formed when a patient discharged from hospital group *H*_*i*_ was admitted to hospital group *H*_*j*_ within a specified time window, *w* (set at 182 days^9,32^); this may be a direct transfer or with an intervening community period. There were 12,062,972 patient movements meeting these criteria, of which 1,225,244 movements (10.2%) represented admissions to different hospital groups. These formed a dense network comprising a single strongly connected component with 137 hospital groups as vertices and 14,986/18,632 (80.4%) of all possible edges between different hospital groups were present. Figure 2 shows the network (without direction) geographically and diagrammatically, with darker lines indicating higher numbers of patient movements. Compared with networks identified from three years’ data pre-pandemic (ending 31 March 2017, 2018, 2019), numbers of hospital groups (range 148–153), non-readmission movements (1,403,711–1,343,625) and density (81.9–82.8%) were lower post-pandemic.

### Simulating novel strain spread

We ran 100 simulations of inter-hospital transmission of strain X from each possible index host facility (all acute hospital groups, *n* = 137), hence 13,700 simulated outbreaks. Transmission simulations used a discrete-time stochastic hospital group-based compartmental model where hospital groups had either *Susceptible* (S) or *Affected* (A) status with respect to strain X, with the pathogen transmitted along the network edges (details in ‘Methods’). We adjusted the network edge weights (i.e. total number of patient movements) by the estimated national prevalence of *C. difficile* carriage^33^. In parallel with this transmission modelling, we simulated the first detection of strain X at each hospital group from CDI case specimens supplied for sequencing. We assumed total CDI cases at each hospital group matched mean monthly CDI case^34^ numbers from mandatory surveillance^35^ and applied a sampling protocol to select sequenced specimens, with strain X detected at an *Affected* hospital group when at least one strain X case was included in those specimens (details in ‘Methods’). We applied two candidate sampling protocols: specimens supplied from all CDI cases or from 10 randomly selected cases per month (per hospital group). From each simulation, we captured the times when each hospital group was affected by strain X, and the corresponding first detection times were that group to supply specimens as a WGS sentinel site.

**Fig. 3:**
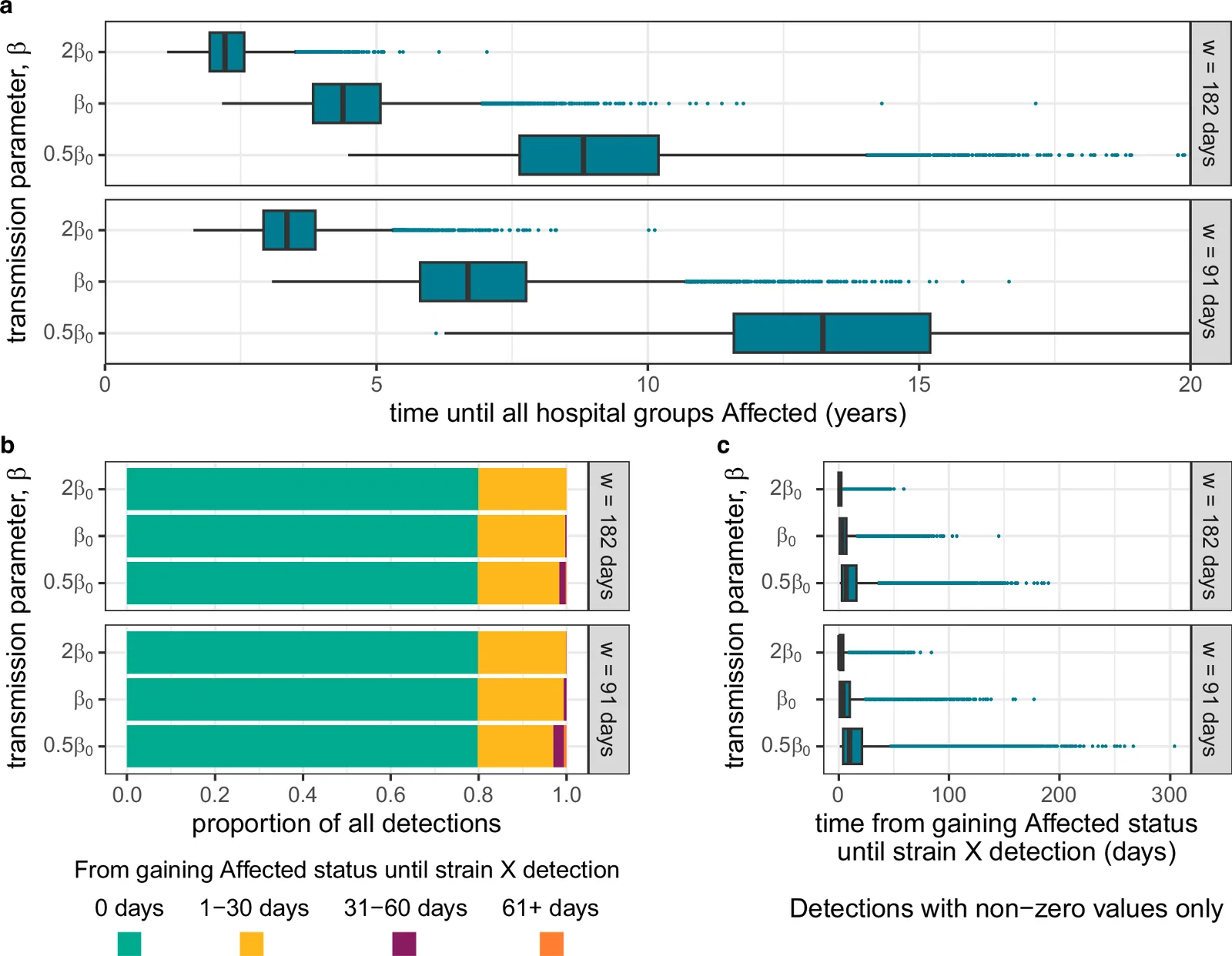
Spread and detection of a novel strain (strain ‘X’). Under different assumptions (transmission parameter, β, and window between inpatient hospital stays, *w*): **a** time (years) from index case seeding until all hospital groups have *Affected* status (*n* = 13,700 simulations each) (**b**) time (days) after hospital group gained *Affected* status until strain X detected there under the 10 specimens per month protocol, all hospital groups*: proportion of all detections with 0, 1–30, 31–60 and 61+ days delay (*n* = 1,876,900 detections each). **c** time (days) after hospital group gained *Affected* status until strain X detected there under the 10 specimens per month protocol: distribution for non-zero values only (*n* = 377,478 to *n* = 379,407 detections each, see (**b**) *Hospital groups with ≤10 *C. difficile* infection (CDI) cases per month always have no delay between a hospital group gaining *Affected* status and strain X detection, by assumption, under this protocol (see Supplementary Fig. 3b). N.B. There is always no delay between a hospital group gaining *Affected* status and strain X detection, by assumption, under the all-cases protocol. Note: boxplots show median, interquartile range (IQR), whiskers (in black) at 1.5 IQR and outliers (in teal), 100 simulations per index case facility (each of the 137 acute hospital groups in financial year FY2022/23 network), detections at each hospital group in each simulation. Censored data for incomplete process at 7300 days: **a** 10 simulations (0.5β_0_, 182 days) and 555 simulations (0.5β_0_, 91 days), (**b**) 11 detections (0.5β_0_, 182 days) and 1280 detections (0.5β_0_, 91 days). Source data are located in the source data file.

All possible index facility seeding locations resulted in strain X spread to all hospital groups, at a rate that depended on both transmission parameter *β* and index facility position within the network. For our base analysis, we calibrated the transmission parameter to a value (*β*_*0*_) with a mean time to affect all hospital groups of 4.5 years (Fig. 3a), consistent with observations from the emergence of RT027 *C. difficile*^3,16^. Doubling this parameter reduced the mean to 2.3 years, whereas halving it increased it to 9.1 years. Halving the period over which patients could carry the pathogen to a subsequent facility (i.e. simulations which used networks constructed with *w* = 91 days), increased the mean time to affect all hospital groups to 6.9, 3.5 and 13.7 years, respectively (i.e. with *β*_0_, 2*β*_0_, 0.5*β*_0_). In the base analysis, 79.8% of detections (*n* = 1,876,900) occurred at the same time under both candidate sampling protocols (79.8–79.9% under alternative {*β*, *w*} combinations) (Fig. 3b).

### Selection of sentinel sites and sampling protocol

Using the detection times from the base inter-hospital spread simulations (with *β* = *β*_0_, *w* = 182 days), we applied a ‘greedy algorithm’ to iteratively build a priority list of sentinel sites, namely at each step selecting from remaining candidate hospital groups to minimise time from strain X seeding until its first detection in any sentinel site (details in ‘Methods’). In the base analysis, candidate sentinels were unrestricted other than excluding specialist hospital groups, which were retained as possible index facilities and could be on the pathway for inter-hospital spread; hence, the algorithm generated a priority list of the 121 non-specialist hospital groups under each sampling protocol. We also incorporated the ability to amend the list of candidate hospital groups at each algorithm step to dynamically respond to existing priority list selections (e.g. to ensure representation by region or hospital group type).

**Fig. 4:**
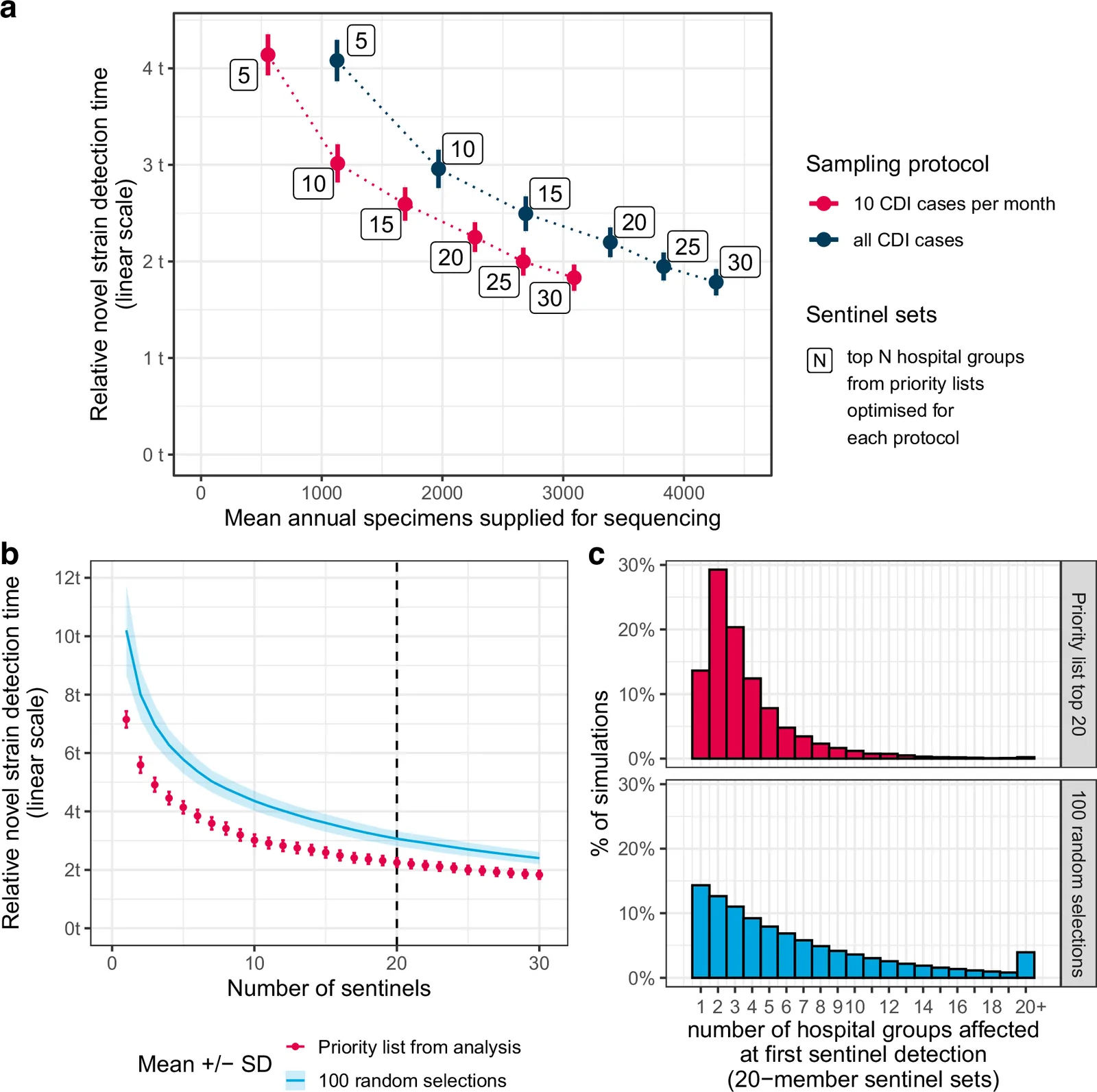
Performance of prioritised sentinel sets. **a** Mean (±standard deviation (SD)) detection time and mean* specimen requirements using specified sizes of prioritised sentinel sets for each candidate sampling protocol (**b**) Mean ( ± SD) strain X detection time sentinel sets of the priority list top 20 (set S_0_) and 100 randomly selected 20-member sets. **c** Number of hospital groups affected when strain X was detected by 20-member sentinel sets: the priority list top 20 (set S_0_) and 100 randomly selected sets. Note: all results based on 13,700 simulations, parameters *β* = *β*_0_, *w* = 182 days (*w*, window between inpatient hospital stays); strain X detection time by a sentinel set is the time from the index case seeding until the earliest detection by any sentinel site(s); detection times are quoted relative to median detection time ‘*t*’ using set *S*_0_ and 10 specimens per month protocol. In (**b**, **c**), the sampling protocol was 10 *C. difficile* infection (CDI) cases/month and random selections excluded specialist hospital groups. *See Supplementary Fig. 1 for distribution of annual specimen counts per protocol. Source data are located in the Source data file.

Comparing 20-member sentinel sets (i.e. the top 20 hospital groups from the priority list generated by the greedy algorithm) constructed under each sampling protocol, the 10 specimens/month protocol generated 33% fewer mean specimens for sequencing per annum than sequencing all CDI cases in sentinels (2269 vs 3390) but expected detection time was only 2% slower (Fig. 4a). The 95% quantile of the estimated annual specimens represented 14.9% or 22.9% of national CDI cases in FY2022/23^35^ respectively. Incremental reductions in detection time decreased progressively as the number of sentinels increased (Fig. 4b): a 20-member set provided an acceptable trade-off between detection time and logistical considerations. The mean first detection time using the priority list’s top 20 as a sentinel set was 27% quicker than using randomly-selected sets of 20 hospital groups under the 10 specimens/month protocol (Fig. 4b) and 28% quicker under the all-cases protocol (100 random set selections, each 13,700 simulations). We therefore considered the top 20 hospital groups on the priority list constructed under the 10 specimen/month protocol (using the base inter-hospital spread simulations) as our benchmark sentinel set (*S*_0_). Using this set, over 13,700 simulations, a mean 3.6 hospital groups had been affected when the strain was first detected (including the index case facility and detecting sentinel, if different) (Fig. 4c), and the source that created the widest spread evading sentinel detection had affected a mean 6.5 other hospital groups before sentinel detection (100 simulations).

### Sentinel set evaluation: sentinel set characteristics

Considering *S*_0_’s composition, regional representation (desirable for a national sentinel programme) was satisfactory, with two or more hospital groups from each of the seven NHS regions included. In terms of hospital group types, *S*_0_ contained no specialist hospital groups (by design), and the majority were teaching hospital groups. Non-use of specialist hospital groups as sentinels was desirable, as these are atypical; constructing alternative sentinel sets relaxing this exclusion, had limited impact on the sentinel set and detection time (highest specialist 18th on priority list). Teaching hospital groups form a minority nationally (48/121 of non-specialists), but constructing alternative priority lists that capped numbers of teaching hospital groups compromised regional representation (one region had a single hospital group in the top 20) and increased detection time vs the benchmark *S*_0_, although the latter could be mitigated by increasing the number of sentinels (Supplementary Fig. 2).

### Sentinel set evaluation: sensitivity to novel strain assumptions

**Fig. 5:**
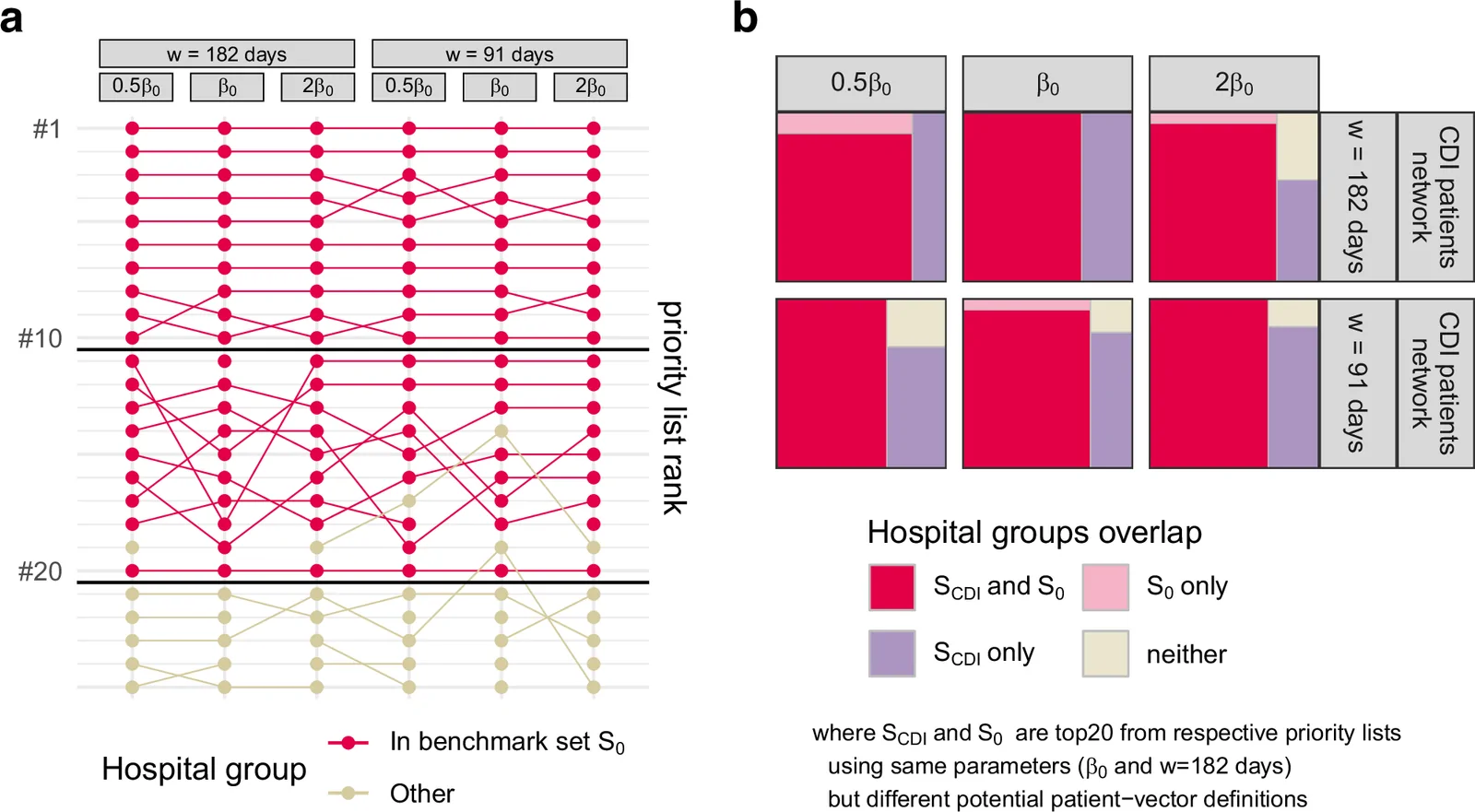
Sensitivity analyses of the benchmark sentinel set S_0_ (novel strain assumptions). **a** sensitivity to transmission parameter, *β*, and window between inpatient hospital stays, *w*: position of specified hospital groups in priority lists for each {*β*,*w*} combination; each line links the positions occupied by a hospital group (**b**) sensitivity to potential patient-vector definition, transmission parameter, *β*, and window between inpatient hospital stays, *w*: top20 composition comparison (vs benchmark set (*S*_0_) and vs ‘*S*_CDI_’) for priority lists for each {*β*, *w*} combination using CDI patient networks; area is proportional to number of overlapping hospital groups Note: **b** CDI patient networks use patient admissions restricted to those with a history of *C. difficile* infection (CDI); ‘*S*_CDI_’ is the CDI patient network equivalent of *S*_0_ i.e. 20-member optimised set, from simulations using same transmission parameter (*β*_0_) and window between inpatient hospital stays (182 days) as for *S*_0_.

There is inherent uncertainty regarding transmission properties of a possible novel strain versus the calibrating strain (itself with limited data), so sensitivity analysis assessed the stability of sentinel prioritisation to variation in the transmission parameter, *β*, and duration of transmissibility (represented by window *w* in the network identification). We determined a plausible range for *β* from our exploratory calibration simulations: the range of timescales compatible with RT027 spread occurred at the extremes of the distributions of simulated observations for *β* within 0.5*β*_0_ to 2*β*_0_ (Fig. 2a). We therefore constructed alternative optimised priority lists using data from strain spreads with *β* = 0.5*β*_0_ and 2*β*_0_. A further three lists (with 0.5*β*_0_, *β*_0_, 2*β*_0_) were constructed using data from simulations where network edges used patient movements from a shorter window (*w* = 91 days), to allow for strains with more rapid reduction in transmissibility and/or carriage. The benchmark set S_0_ overlapped largely with the 20-member sets constructed from these alternative priority lists (Fig. 5a), with identical hospital groups occupying the top 10 positions (albeit in different orders) and 18/20 of *S*_0_ members present in all lists. The detection time advantage was retained for prioritised list sentinels vs random sentinels across all scenarios, with reduction in mean detection time ranging from 27 to 29%.

We repeated analyses with a stricter definition of patients who could act as possible vectors, to allow for a strain where transmission focussed on patients with a CDI history. We linked patient movement records with mandatory CDI surveillance data (matching on patients’ NHS number, hospital number and date of birth) and simulated strain X transmission across the network with edges weighted by movements of CDI patients only. The top 10 members of the benchmark priority list and the list obtained restricting to CDI-history patients (using the same parameters, *β*_0_ and *w* = 182 days) largely overlapped (9/10 present), but thereafter diverged more (14 hospital groups present in both top 20 s) (Fig. 5b). Across the same sensitivity analysis (${\beta }\in \left\{{0.5{\beta }}_{0},{\beta }_{0},{2\beta }_{0}\right\},w\in \{182,\,91\}{{{\rm{days}}}}$), most hospital groups (15) were present in all top 20 s, and all but one had the same top 10 members. In that exception, the top priority hospital group changed vs S_0_ to another from the same region; applying the greedy algorithm with a forced first choice matching S_0_ reordered, but did not change, the hospital groups in the rest of the top 20, suggesting these hospital groups performed similar roles within this patient movement network.

### Sentinel set evaluation: stress-testing for changes over time

Sentinel surveillance system performance needs to remain robust to the unknown future trajectories of CDI incidence and secondary care use.

**Fig. 6:**
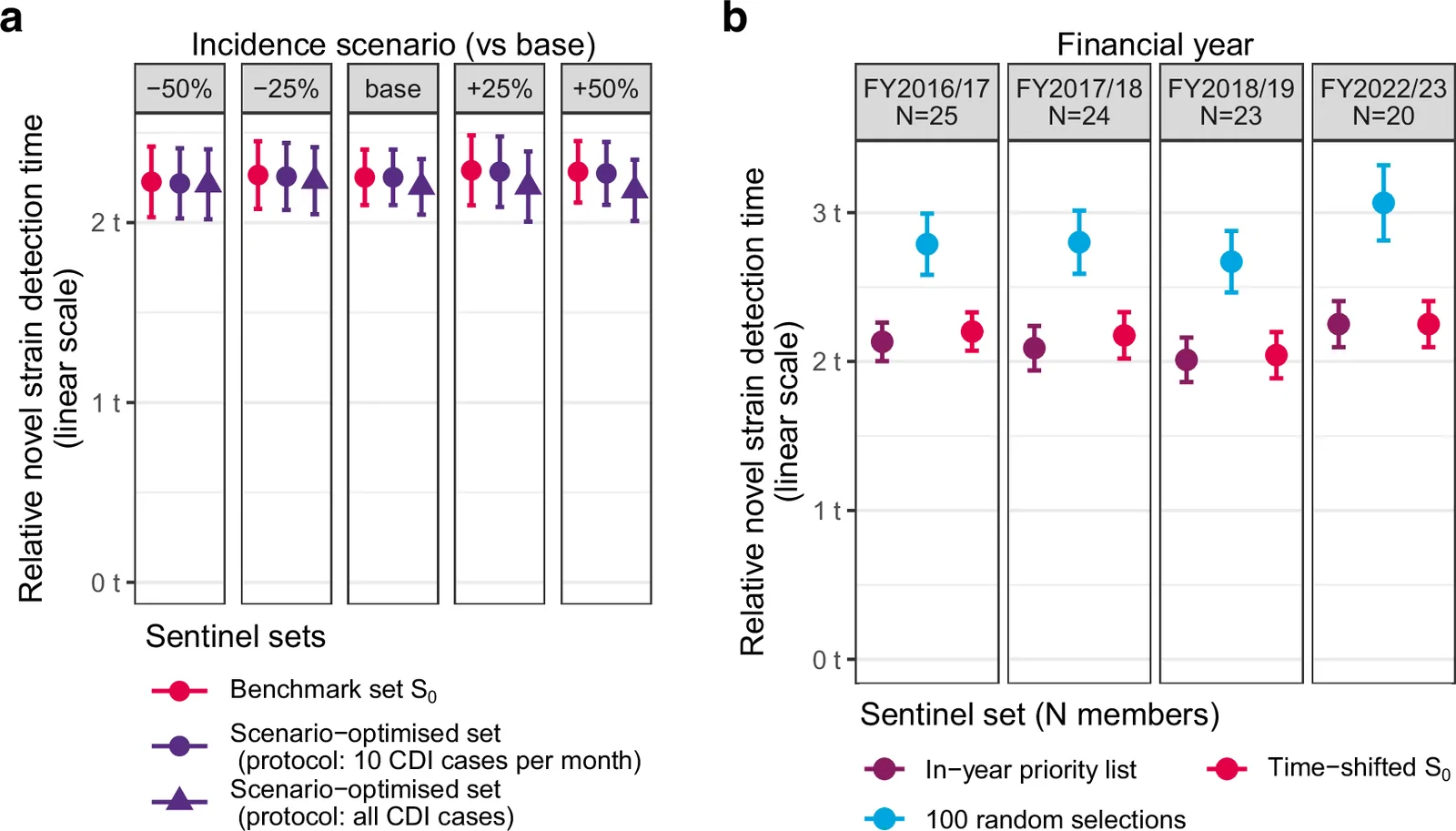
Scenario analyses of the benchmark sentinel set *S*_0_ (temporal variation). **a** *C. difficile* infection (CDI) incidence scenarios: Mean (±standard deviation (SD)) detection times of benchmark sentinel set *S*_0_, sentinel set optimised for incidence scenario, sentinel set optimised for incidence scenario using alternative sampling protocol (sequence all CDI cases); all sets with same size (20) and same simulation parameters (*β* = *β*_0,_ *w* = 182 days) as for *S*_0_ (**b**) in-the-past scenarios: Mean (±SD) detection times of benchmark sentinel set S_0_ (time-shifted from financial year FY2022/23 to each year), in-year prioritised sentinel set and 100 randomly selected sets of N hospital groups, where N is the size of the time-shifted *S*_0_ set; all sets with the same simulation parameters as for *S*_0_ (*β* = *β*_0,_ *w* = 182 days). *w* window between inpatient hospital stays Note: all results based on 13,700 simulations per scenario; detection times are quoted relative to median detection time ‘*t*’ using set *S*_0_ and 10 specimens per month protocol in financial year FY2022/23. **a** Network and transmission as for *S*_0_ in all scenarios, base scenario uses empirical multi-year mean reported CDI cases, (**b**) uses in-year simulation data, within each year the compared sets are same-sized (25, 24, 23, 20, respectively, given historical mergers affecting *S*_0_). FY2016/17 uses the network of prior movements for admissions between 1 April 2016 and 31 March 2017. FY2017/18 uses the network of prior movements for admissions between 1 April 2017 and 31 March 2018. FY2018/19 uses the network of prior movements for admissions between 1 April 2018 and 31 March 2019. FY2022/23 uses the network of prior movements for admissions between 1 April 2022 and 31 March 2023. Source data are located in the Source data file.

We therefore first simulated detection of strain *X* using *S*_0_ in scenarios with a range of overall CDI incidence vs the multi-year average used in the base analysis (uniformly scaling all hospital group case numbers by 0.50, 0.75, 1.25 and 1.50) and applying both candidate sampling protocols. In all scenarios (using ${{\beta }}_{0}$ and *w* = 182 days), *S*_0_ detected strain X in comparable times for each candidate protocol (Fig. 6a). Both incremental detection time and difference in sequenced specimens for *S*_0_ vs scenario-optimised sets sequencing all cases increased with higher incidence (0%, 1%, 4%, 5% slower and 0%, 17%, 40% 52% fewer, respectively) as more hospital groups exceeded 10 cases/month (Supplementary Fig. 3). Most *S*_0_ members were also in the top 20 of priority lists optimised within each scenario (at least 17/20 identical), supporting robust sentinel selection across ±50% overall CDI incidence (but noting significant incidence changes may prompt re-analysis regarding WGS capacity).

Finally, we stress-tested the performance of S_0_ if used in the past as a substitute for future generalisability. Using base case *β*_0_ and *w*, simulations of strain X spreading across networks built for each of the last three years pre-pandemic (FY2016/17, FY2017/18, FY2018/19) had comparable mean times to spread to all hospital groups (4.5, 4.3, 4.1 years respectively; from 15,300, 15,100, 14,800 simulations respectively). Most (75–80%) *S*_0_ members (or their predecessors, if not existing in their present form) were also present in the top 20 in-year priority lists (determined from each year’s simulations using the greedy algorithm with the same criteria as *S*_0_). For each year, we created time-shifted versions of *S*_0_, replacing set members with their predecessors^36^, including both components before any organisational mergers (resultant set sizes 25, 24, 23), and determined the mean time to first detection for the time-shifted *S*_0_, 100 same-sized contemporarily-selected random sets and for an in-year optimised set (same number, taken from the in-year priority list) (Fig. 6b). The time-shifted S_0_ continued to outperform random selections in all years. The mean first detection by each time-shifted *S*_0_ was slower than by the in-year optimised sets, but these differences were not significant. Similar relative performance was observed with equivalent analyses using 0.5*β*_0_ and 2*β*_0_ (Supplementary Fig. 4). Without future data on patient movements or changes in service provision by hospital group, this retrospective analysis supported acceptably robust sentinel selection over a five-year timescale with similar types of temporal changes in secondary care provision and use.

## Discussion

Selection of which specimens to sequence is a key step in the design of genomic surveillance^37,38^; here, through both choice of sentinel sites and sampling protocol within sentinel sites, we have leveraged the limited laboratory capacity available for introducing *C. difficile* WGS surveillance to enable early, efficient detection of hypothetical novel strains. The set of 20 sentinel hospital groups identified is predicted to rapidly intercept the inter-hospital spread of a novel strain across a broad range of potential transmission characteristics and irrespective of index case location: the network-analysis-informed site choices maximise the system-wide insight available from a few sentinels. We have achieved this without additional patient sample collection; however, implementing a new surveillance system requires investment in people, resources and systems, which may prompt real-world considerations beyond laboratory capacity: in this analysis, sentinel set selection was satisfactorily robust to temporal variations at pathogen and healthcare system scales. We have also demonstrated how the methodology provides evidence on best adjustments (to still meet the goal of rapid detection) if circumstances require some flexibility or external constraint on sentinel selection.

We achieved this by extending previous work^24^ in multiple dimensions, introducing separate (but linked) timelines for pathogen spread and pathogen detection, dynamically-defined candidates within the existing ‘gold standard’^24,25^ selection algorithm to incorporate a variety of real-world constraints, network analysis of linked data for patient movement and infection cases, estimation of uncertainty in sentinel set detection time, and structured stress-testing of proposed sentinel sets including measuring detection performance in different contexts than those used in set derivation.

We used a parsimonious model simplifying complex mechanisms, operating at scales from within-host to inter-hospital group, that underlie a hospital group gaining *Affected* status and the number of CDI specimens available for sampling in each hospital group. These simplifications enabled multiple intermediate, inherently unknown parameters to be conflated into a limited set, which we were able to manipulate to test sentinel performance under a wide range of possible pathogen characteristics and future surveillance contexts.

Simplification regarding change of hospital group status is discussed in detail in Ciccolini et al.^24^ and van Bunnik et al.^25^. For clarity, we repeat their observation^24^ that a hospital group may legitimately be a multi-hospital organisation, in which case *C. difficile* presence might vary across component hospitals, just as within a single hospital, it can vary across wards. However, in England, groups without a sole or dominantly large acute hospital form the minority^39^. Additionally, these hospital groups, the institutions responsible for acute care in NHS England, are also the units for CDI mandatory reporting and accountability^30,31^; hence, using them as the surveillance unit is a natural extension, and assumption of within-group homogeneity is a reasonable model simplification. Key assumptions underpinning our simulations of pathogen spread are relatively limited community transmission (at least of any novel strain), and that *Affected* hospital groups remain *Affected* and capable of onward strain X exportation via discharged inpatients. *C. difficile* natural history^9^ and evidence from RT027’s spread^16,17^ suggest these are reasonable. In England, relatively higher community contribution to CDI cases since RT027’s decline^40^ (possibly due to successful hospital-based interventions^41^) may have peaked^26,27^, and studies in other countries have found good agreement between network-model-derived incidence predictions and reported CDI cases by facility^23,25^. We note that these assumptions mean this methodology will be most useful for strains with stronger sustained transmission, which include the operationally concerning strains that the new WGS sentinel system is tasked with discovering.

Detection based on sampling cases was not considered in Ciccolini et al.^24^ nor other similar studies comparing simulation-informed hospital-based surveillance designs^8,25,42^, so we discuss our related assumptions in more detail. We assume that detection is only possible once a hospital group has *Affected* status (i.e. there is sustained transmission), hence exclude possible detection from any CDI cases prior to this, which do not result in onward strain X transmission; we assume this would not occur differentially by hospital group, which might affect sentinel selection. Our model assumes all CDI cases have specimens taken (consistent with findings for England^43^) and that sequenced specimens are a random sample. Mandatory reporting data were used to calibrate total CDI cases (rather than using simulated values from within-organisation models), and we employed parsimonious parameterisation to determine strain X’s attributable cases, using the continuing status-change force from the hospital group status process. This simple approach is reasonable given most acute hospital groups (75%) have ≤10 mean monthly cases^35^, hence specimens from any CDI case would be sequenced under both candidate protocols. The proportion of *C. difficile* colonisations which are symptomatic varies by strain^44,45^ and might be expected to higher for our target strains of greater concern. In this case, the model would disproportionately underestimate detection time in higher incidence hospital groups; the benchmark sentinel set is thus advantageous as higher incidence hospital groups are well-represented. Within the stress testing, we applied changes uniformly across empirical total CDI cases; therefore, we cannot estimate the impact on strain X detection if strain-mix differentially affects total cases at a hospital group-level granularity. Finally, this analysis does not consider factors beyond specimen supply; we assume sentinel selection is equitable regarding sequencing technique and costs, turnaround time, and across other implementational considerations for WGS and surveillance systems more generally^37,46^.

Limitations include that data available for network construction were constrained by the SARS-CoV-2 pandemic, during which admissions and patient movements were atypical^47,48^ and which may have prompted residual changes^49^; complete data for analysis were available for one post-pandemic year only (i.e. after legal movement restrictions were removed^50^). This is mitigated by the robust performance of the benchmark sentinel set when applied retrospectively, for both composition and performance metrics. CDI case data at hospital group granularity are vulnerable to temporal fluctuation from local outbreaks; hence, multi-year averages were used in preference to single-year data (in modelling sampling protocols and sample burden estimation). This was not possible with the linked CDI case movement data, but these were only used in one sensitivity analysis, limiting potential influence on the overall results. We observed differences in network characteristics pre- vs post-pandemic, but it is not yet clear if this is a pandemic legacy or due to typical ongoing changes of secondary care provision^51^, nor if any changes are temporary or long-lasting. Hence, once more post-pandemic data are available, robustness will be re-tested as part of post-implementation service evaluation, which will also consider a formal cost-analysis (subject to appropriate empirical data).

Due to ad-hoc or incomplete ribotype reporting, especially prior to the *C. difficile* Ribotyping Network introduction in 2007 (believed to be after the peak in RT027^52^), calibration data for rate of strain spread were limited. We therefore estimated a plausible range for novel strain transmission, although we were unable to adjust for any confounding from differences between contemporary networks vs those in place when RT027 cases were rising and for the impact on transmission from temporal differences in infection prevention and control (IPC) practices or relating to variation in CDI testing vs current guidance. For the duration of each simulation (up to 20 years), we assumed an invariant network, i.e. one of the static networks constructed from different cross-sectional patient movement datasets, and invariant transmission properties. These assumptions are not plausible across the full duration modelled, but this simulation length is necessary only to provide the selection algorithm with average values which are minimally affected by censoring; in reality, we only require these assumptions to hold until first detection (hence also before any reactive IPC changes), which is a more reasonable timescale for invariancy.

Expanding WGS to the surveillance of key healthcare-acquired infections is recommended by supranational organisations such as the European Centre for Disease Prevention and Control^53^ to facilitate detection of emergent high-risk strains/plasmids/variants. The sentinel selection methodology presented here could be applied in different geographies and to other pathogens which have predominantly hospital transmission (of a novel strain) and for which patients can act as inter-hospital vectors, subject to data availability (network generation and models’ parameterisation/calibration) and case-by-case consideration of model assumptions. This may include key antimicrobial resistance threats such as CPE or MRSA in some geographies. We were agnostic to novel strain index facility, simulating all possible hospital groups equally in this role, but any intelligence on likely origins (e.g. cross-border patients) could be incorporated by reproducing the risk profile in the proportion of simulations with each index facility, which would then provide a weighted dataset of spread patterns. Reviews of published shared patient networks^5–7^ found heterogeneity in network characteristics (albeit across many network inclusion definitions): our networks were all single-component, strongly-connected networks, but weakly connected or multi-component networks would require piecewise analysis (as full network spread would be impossible from some index facilities). We had satisfactory consensus on sentinel sets across our sensitivity analyses and stress-testing: other pathogens/geographies may not have clear consensus, but our approach of stress-testing across a range of futures is easily extended to a formal decision analysis, such as in the framework of Decision Making Under Deep Uncertainty^54^ to identify a robust, well-performing (if not optimal) sentinel selection.

In conclusion, this analysis has provided a stress-tested surveillance sentinel set to deliver rapid, efficient identification of novel *C. difficile* strains within real-world logistical constraints that has been launched in England in 2025 to enable prompt implementation of infection control and treatment options to reduce the potential impact on healthcare provision from these threats. It also provides a roadmap for future hospital-acquired pathogen surveillance.

## Methods

### Ethics statement

No ethical approval was sought for this study as UKHSA has approval under Regulation 3 of the Health Service (Control of Patient Information) Regulations 2002 to process national surveillance data, containing patient-identifiable information, without consent.

### Identification of inter-hospital network of patient movements

We identified directed, weighted networks of inter-hospital patient movements from empirical inpatient data. Network vertices were NHS acute hospital trusts (trusts are secondary/tertiary care organisational units within NHS England, subsequently denoted hospital groups). A directed network edge, *H*_*i*_ → *H*_*j*_, was formed when a patient discharged from hospital group *H*_*i*_ was admitted to hospital group *H*_*j*_ within a specified time window, *w*; edges thus consist of both direct transfers and admissions with an intervening community period. The number of these patient movements was used in weighting the network edge (see below).

We identified the prior movement associated with all hospital stays (including multiple per patient) with admission dates during 1 April 2022–31 March 2023 (denoted FY2022/23, first full year of data available entirely after the SARS-CoV-2 pandemic) with time window *w* = 182 days, i.e. the patient had been discharged from their previous hospital stay at any point up to 182 days before the admission date. The full dataset interrogated thus included hospital stays with either discharge dates during 1 October 2021–31 March 2023 or admission dates in FY2022/23, to allow for a moving 182-day window for each possible admission date. The maximum 182-day timespan between hospital stays was informed by studies of patients newly asymptomatically colonised with *C. difficile* whilst in hospital^9,32^ and of *C. difficile* carriage/excretion duration within the community^9^. This post-pandemic period was after the relaxation of the final government-mandated community-based measures, which would necessarily affect the movement of potential vectors^50,55^; we assume patients were thus able to resume a ‘normal’ pattern of hospital attendance. Inpatient data were extracted from Hospital Episodes Statistics (HES) Admitted Patient Care database^28^ in the form of ‘consultant episodes’ (periods of hospital care). A hospital stay may be reported as multiple consultant episodes; we defined a single continuous hospital stay as overlapping consultant episodes or an episode ending with a reported discharge to another within 0–2 days, for the same patient occurring in the same hospital group. We identified episodes for the same patient by stepwise record linkage using combinations of patients’ NHS numbers, hospital numbers, and date of birth. We included only stays in acute hospital groups (as included in the mandatory CDI case reports^35^, with further hospital group characteristics determined from Estate Returns Information Collection reports (ERIC)^39^), and if there was organisational change during the data period (e.g. mergers), we reallocated records to the hospital group as at 31 March 2023^36^.

The analysis was performed using R version 4.4.1^56^. We used the HospitalNetwork R package^29^, which builds on several earlier studies^24,42,57^, to construct the network from these hospital stay data. In the base analysis, we then adjusted each edge-weight (the number of patient movements) by estimated prevalence of *C. difficile* carriage (using a national estimate, 3%^33^, in the absence of more granular data due to the symptomatic criteria required for testing^34^ and reporting^30^) to scale to numbers of possible novel-strain-carrying vectors; hence the base analysis adjacency matrix has elements:

1 $${M}_{i,j}=0.03{m}_{i,j}$$

where ${m}_{i,j}$ is the number of patient movements from *H*_*i*_ → *H*_*j*_. We also constructed networks for pre-pandemic years (FY2016/17, FY2017/18, FY2018/19), for a shorter window (*w* = 91), and with edge-weights scaled to patients with a history of CDI (reported during any hospital stay during the 18 months’ data for each year). For the latter, all CDI cases in England (for all the years covered by the hospital movement data) were extracted from the Healthcare-associated Infection Data Capture System^58^ and deterministically linked to the admission dataset by stepwise linkage using the same combinations of patients’ NHS number, hospital number and date of birth. The CDI patient networks were constructed from the hospital stay data restricted to movements by patients with a CDI history, and the adjacency matrix elements were the network edge-weights (the number of CDI-history patient movements) without further adjustment.

### Simulation of inter-hospital spread of a novel strain

We simulated the inter-hospital spread of a novel *C. difficile* strain (‘strain X’), with the pathogen transmitted via the edges of the patient-movement-defined network of acute hospital groups.

The infection transmission simulations used a discrete-time stochastic hospital group-based compartmental model, in which hospital groups have either *Susceptible* (S) or *Affected* (A) status with respect to the novel strain. Once a hospital group is *Affected* it retains this status indefinitely (through sustained transmission between patients and/or from environmental *C. difficile* spores) and is capable of onward transmission. As in Ciccolini et al.^24^, we combine the complex steps for transition S → A into a single transmission parameter, *β*, applied to the weights of the incoming network edges from *Affected* hospital groups. At each time step, the probability of a *Susceptible* hospital group, *H*_*j*_, gaining *Affected* status is given by:

2 $${P}_{{H}_{j}\to {AFFECTED}}=1-\exp \left(-\beta {\sum }_{{H}_{i}\in \left\{A\right\}}{M}_{i,j}\right)$$

where *M* is the adjacency matrix for the inter-hospital movement network, with edge-weights scaled for *C. difficile* carriage or CDI history as appropriate for the scenario, and {A} is the set of *Affected* hospital groups. We assumed all acute hospital groups could be the index facility for a novel strain (with equal probability), so for each spread scenario (network and transmission combination), we performed 100 simulations with each of the acute hospital groups as index location. Simulations ran until all hospital groups were *Affected* and had detected strain X under both candidate sampling protocols, or 7300 days (whichever was sooner). We used Ciccolini’s method to calibrate *β* for the base analysis, here comparing the distribution of time until all hospital groups obtained *Affected* status (across all the simulations, including every hospital group as index facility location) with the timescale of the *C. difficile* RT027 epidemic in England^3,16^. This approach implicitly incorporates the effect of the overall distribution of carriage duration (censored at the window length *w*), despite the lack of explicit granular data on carriage prevalence and duration.

### Simulation of novel strain detection

After each hospital group gained *Affected* status, we simulated the time until the novel strain would be first detected there (should it be selected as a sentinel), i.e. a specimen from a CDI case attributable to strain X was sampled for sequencing. The sampling protocol was applied to total annual CDI case numbers for each hospital group taken from empirical data, using a multi-year average to minimise impact from short-term local outbreaks.

Total monthly CDI cases for each hospital group were fixed at their three-year mean value from mandatory CDI surveillance (FY2016/17-FY2018/19, selected as a non-pandemic period with consistent reporting criteria and stable national case count)^35^; we assumed all CDI cases were reported in these data^43^ and would have specimens available. National guidance on CDI case diagnosis^34^ was invariant across analysis period, with evidence of widespread local implementation^59,60^. This recommended testing of patients with CDI symptoms (diarrhoea) using a two-stage testing approach (a glutamate dehydrogenase (GDH) enzyme immunoassay (EIA) test followed by a sensitive toxin EIA test); screening of asymptomatic patients for *C. difficile* carriage was not recommended. Where hospital groups had merged/demerged^36^, we weighted reported data by the proportions of constituent hospital groups in the combined entity. We simultaneously simulated detection under two candidate sampling protocols. Under a protocol to supply specimens from all CDI cases for sequencing, we assumed a strain X case specimen was supplied at the same time step as the change in status. The alternative sampling protocol was based on instructions for sentinel hospital groups to supply specimens from 10 randomly selected CDI cases per month. Hence (via the hypergeometric distribution) the probability that these *n* = 10 specimens all fail to include a strain X case is given by:

3 $${P}_{{monthly\; fail}}=\frac{\left(\begin{array}{c}{pN}\\ 0\end{array}\right)\left(\begin{array}{c}N-{pN}\\ n\end{array}\right)}{\left(\begin{array}{c}N\\ n\end{array}\right)}$$

where *N* is total CDI cases per month, and *pN* is the strain X CDI cases per month, assumed >0 in *Affected* status hospital groups. The daily probability that a strain X case was included within the specimen(s) supplied for sequencing was modelled as a Bernoulli trial, where the monthly cumulative probability of detection failure has the same value as above (under simplifying assumptions of a 30-day month with constant strain X proportion of all cases), given by:

4 $${P}_{{daily\; detection}}=1-{\left({P}_{{monthly\; fail}}\right)}^{1/30}$$

(A heat map of probability of detection within a month, for combinations of CDI case numbers—total and strain X—is given in Supplementary Fig. 3). We used the continuing transmission pressure from *Affected* hospital groups as the dynamic proportion of strain X attributable cases (rounding to whole number of cases for use in the hypergeometric distribution).

### Prioritisation of sentinel sites for rapid novel strain detection

Using the detection time results from the inter-hospital spread simulations, we used a ‘greedy algorithm’ to iteratively build a priority list of sentinel sites, selecting sites to minimise overall time until detection at each step.

At each iterative step, the prioritisation algorithm used the mean simulated detection times (per index facility) for each hospital group that was a candidate for addition as a sentinel site. The first entry on the priority list was the candidate hospital group with the minimum mean of detection times across all index facility locations. At step *N* + 1 the algorithm took the existing priority list of *N* hospital groups, then added each remaining candidate hospital group, in turn, and calculated the mean (across all index locations) of the earliest mean detection time from that list of *N* + 1 hospital groups. The added hospital group, which resulted in the quickest mean detection time, was selected to add to the priority list (any ties were resolved by random selection from the top-priority hospital groups). i.e. at step *N* + 1, with candidate list $\{{c}_{k}\}$ and existing priority list (of N hospital groups) ${L}_{N}$

5 $$	{{\rm{choose}}}\,{c}_{k}{{\rm{which}}}\,{{\rm{give}}s}\,{{\rm{minimum}}}\,{{\rm{value}}}\,{{\rm{of}}}{\,{{\rm{mean}}}\,}_{i\in \{{H}_{i}\}}({F}_{i,k}) \\ 	 {{\rm{where}}}\,{F}_{i,\,k} ={\min }_{j\in \left\{{L}_{N},\,{c}_{k}\right\}}({D}_{i,\,j})$$

where ${D}_{i,j}$ is matrix of mean detection time at hospital group *j* from simulations with index facility *i*, and $\{{H}_{i}\}$ is the set of all acute hospital groups.

We were able to amend the candidate list of hospital groups at each step, using criteria which could be invariant across all steps or which could be dynamically responsive to nominated characteristic(s) of the existing priority list at each step. This dynamic candidate list mechanism enabled us—if desired—to control the use of specified hospital groups or sub-groups of hospital groups, reflecting real-world concerns such as use of specialist/teaching hospital groups, or to explore the impact of forced inclusion/exclusion of specific locations, within an optimising framework. Independent of any candidate list criteria, all hospital groups were retained as vertices in the network and were able to be on pathways for inter-hospital pathogen spread.

### Sentinel set evaluation: performance, composition and sequencing burden estimation

Prioritised sentinel sets were taken from the priority list obtained for the specified scenario (network, transmission, sampling protocol, and any candidate hospital group constraints), e.g. for a set of 20 sentinels, the top 20 hospital groups on the priority list were selected. First detection time was the priority outcome of interest to evaluate sentinel performance, but absolute detection time is not reported—as this is necessarily related to the (unknown) transmission rate of strain X—instead, we evaluate relative detection time by the prioritised set to detection by comparison set(s).

We estimated the distribution of a sentinel set’s first detection time (for a scenario) by extracting simulation-by-simulation first detection times (i.e. minimum of all sentinel hospital group’s detection times), using 100 simulations per possible index hospital group as above, and calculated summary statistics for this distribution (typically mean and standard deviation). We note that the optimising algorithm used a mean value across simulations, as it required a single value for clear comparison of different candidate hospital groups at each iterative step. A secondary measure of sentinel set performance was how widely the strain had spread before detection, so we extracted the status of all hospital groups at the first detection time, on a simulation-by-simulation basis. Characteristics of hospital groups (NHS region and hospital group type) were taken from ERIC^39^ and used to evaluate the composition of the sentinel set. Comparison sentinel sets with different mixes of hospital group types was obtained by applying the optimising selection algorithm to the same simulated data, but with different candidate hospital group restrictions, e.g. specifying the maximum number of teaching hospital groups to be included in the priority list (once the cap is reached, the remaining teaching hospital groups are excluded from the dynamic candidate list).

For implementation, we required an estimated distribution of annual sequencing specimen burden, for comparison with capacity and to inform surveillance budget estimates. We fitted a normal distribution (with mean 1) to the set of annual indices (i.e. $\frac{{annual}\,{CDI}\,{cases}}{{multi}-{year}\,{mean}}$) for all non-specialist acute hospital groups (data from FY2016/17-FY2018/19 mandatory surveillance^35^, as above), and then applied each hospital group’s mean CDI case count as a rescaling factor to obtain a distribution with hospital group-specific means. For the national burden, we used 100 sum counts of hospital group-by-hospital group counts drawn from these normal distributions, each capped (if required) according to the sampling protocol.

### Additional sentinel sets used in sensitivity analysis and stress-testing

A benchmark sentinel set was identified from the base analysis, with specialist hospital groups excluded from sentinel candidacy.

For sensitivity analyses of the sentinel selection vs strain X transmission assumptions, we constructed comparison sentinel sets from priority lists (with the same exclusion of specialists and same sampling protocols) but using simulated pathogen spread data from alternative parameter assumptions. Specifically, we considered combinations of alternative transmission parameter *β* (plausible values determined from the base analysis calibration process) and a shorter inter-stay window (*w* = 91 days), and using the networks with edge-weight values restricted to patients with CDI history.

To stress-test for changes in overall CDI incidence, we obtained alternative detection times for scenarios with adjusted monthly total CDI case values used in the sampling protocol modelling. We applied a multiplicative factor (in the range 0.5–1.5) to the empirical CDI case dataset, and associated detection times for all adjusted datasets were modelled in the context of the same inter-hospital spread simulations (infection transmission being independent of detection status). These detection time data were used in the construction of priority lists and corresponding sentinel set evaluation to stress test the benchmark set to changes in overall CDI incidence.

To stress-test for temporal changes in patterns of secondary care usage, we generated spread simulation data using networks built using data from the three most recent pre-pandemic years (FY2016/17, FY2017/18, FY2018/19) (with *β* and *w* values as above). These data were used to construct in-year priority lists using similar selection conditions as in FY2022/23. We also obtained time-shifted versions of the benchmark set for each of these years. The time-shifted sets included all those hospital groups whose hospitals were managed by the hospital groups in the benchmark set in FY2022/23, e.g. both predecessors if a benchmark hospital group was formed from a merger of hospital groups active in earlier years^36^, so the time-shifted set could contain a different number of hospital groups to the original. The in-year simulation data were used to estimate the detection performance (as above) of the time-shifted benchmark set, the in-year optimised sentinel set (of the same size) drawn from the in-year priority list and 100 randomly selected sets of hospital groups in each of the three years.

### Reporting summary

Further information on research design is available in the Nature Portfolio Reporting Summary linked to this article.

## Supplementary information

Supplementary Information

Peer Review file

Reporting Summary

## Source data

Source Data

## Data Availability

Hospital Episode Statistics (HES) data^28^ are available from NHS Digital, but restrictions apply to the availability of these data, which were used under licence for this study, and so are not publicly available. NHS Digital’s Data Access Request Service (DARS) provides information regarding the requirements and process to access HES data at https://digital.nhs.uk/services/data-access-request-service-dars, which also includes contact details for enquiries. Data from the mandatory surveillance of *C. difficile* infections^35^ is published, summarised at an NHS acute trust granularity, publicly available from https://www.gov.uk/government/statistics/clostridium-difficile-infection-annual-data. Published data does not include patient-level data (used for linkage with admission data) obtained from the UKHSA’s HCAI data capture system (DCS^58^). Access to these data is restricted due to privacy reasons as they contain personally identifiable information (PII) collected for public health protection and statutory surveillance; queries should be addressed to data.access@ukhsa.gov.uk, cc’ing mandatory.surveillance@ukhsa.gov.uk, with access to anonymised data considered subject to justification for access, relevant governance approval and requiring a data sharing agreement. Source data are located in the source data file. Source data are provided with this paper.

## Code availability

The R code essential for the analysis is available from https://github.com/ukhsa-collaboration/Sentinel_Selection_Using_Network_Analysis. This code uses network information in the form of a HospiNet object, output by the HospitalNetwork package^29^ which may be downloaded from https://CRAN.R-project.org/package=HospitalNetwork.
