# Supplementary material for: Novel surveillance design exploiting network analysis for efficient, rapid detection of emerging Clostridioides difficile strains
Source: Nat Commun. 2026 Aug 26;17:9051. doi: 10.1038/s41467-026-72311-0 (PMC13518965; doi:10.1038/s41467-026-72311-0)
Supplement: Supplementary file 1 [file 41467_2026_72311_MOESM1_ESM.pdf]

Novel surveillance design exploiting network analysis for efficient, rapid detection of emerging *Clostridioides difficile* strains

SUPPLEMENTARY FIGURES

and Reference for Supplementary Figures

Supplementary Figure 1:	Distribution of estimated annual sequenced specimens supplied by 20-member, optimised sentinel sets
Supplementary Figure 2:	Impact of restricting the number of teaching hospital groups on mean time to detection
Supplementary Figure 3:	Probability of detection within a month under protocol sampling 10 CDI cases per month (cf monthly CDI cases per hospital group)
Supplementary Figure 4:	In-the-past scenario analyses using transmission parameters ½β_0 and 2β_0

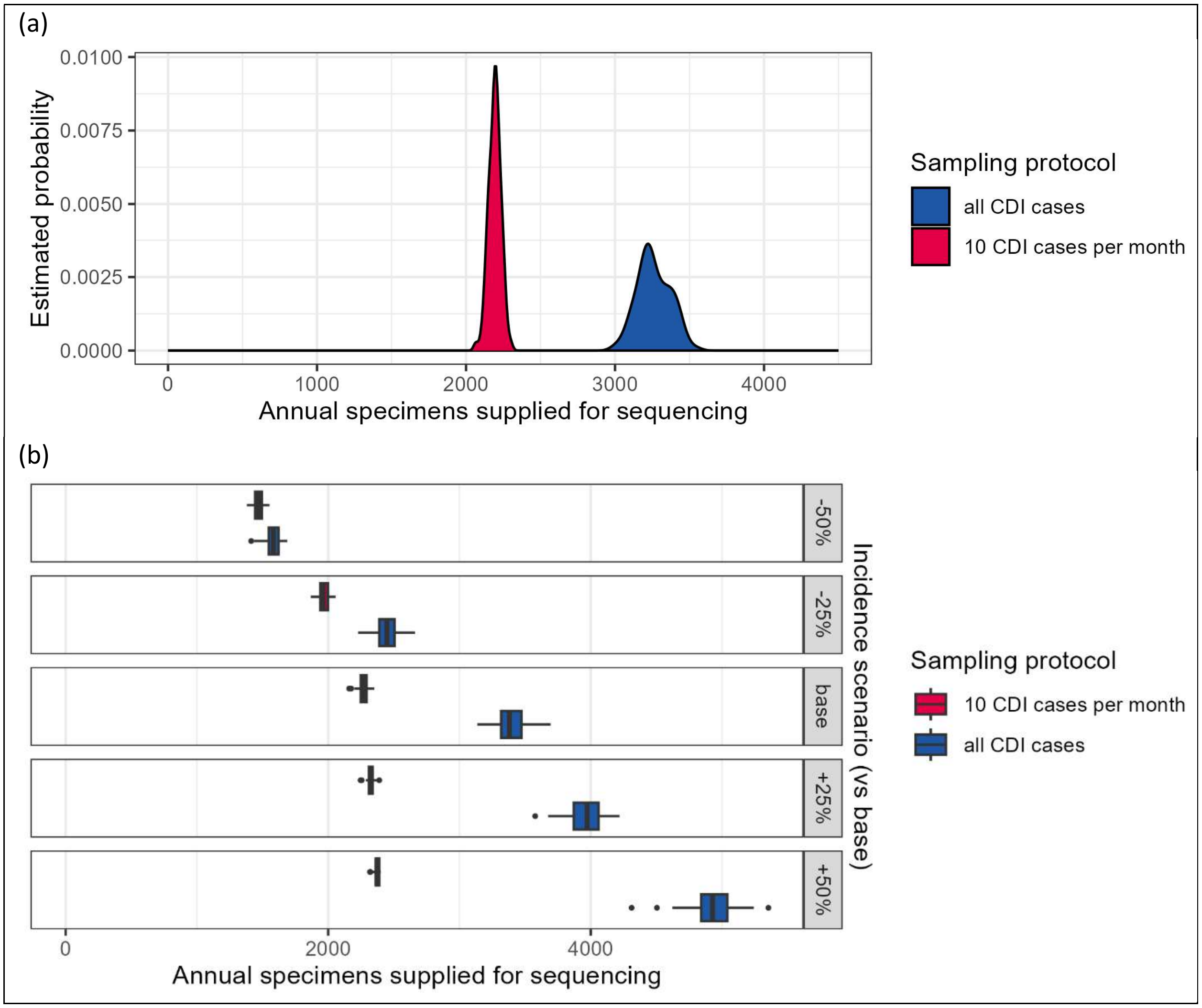

Supplementary Figure 1: Distribution of estimated annual sequenced specimens, supplied by 20-member optimised sentinel sets, under candidate protocols for (a) base analysis and (b) incidence scenarios

Estimated cases by hospital group randomly drawn (n=100) from normal distributions centred on the 3-year mean of reported cases.

Note: (b) boxplots show median, inter-quartile range (IQR), whiskers (in black) at 1.5 IQR and outliers

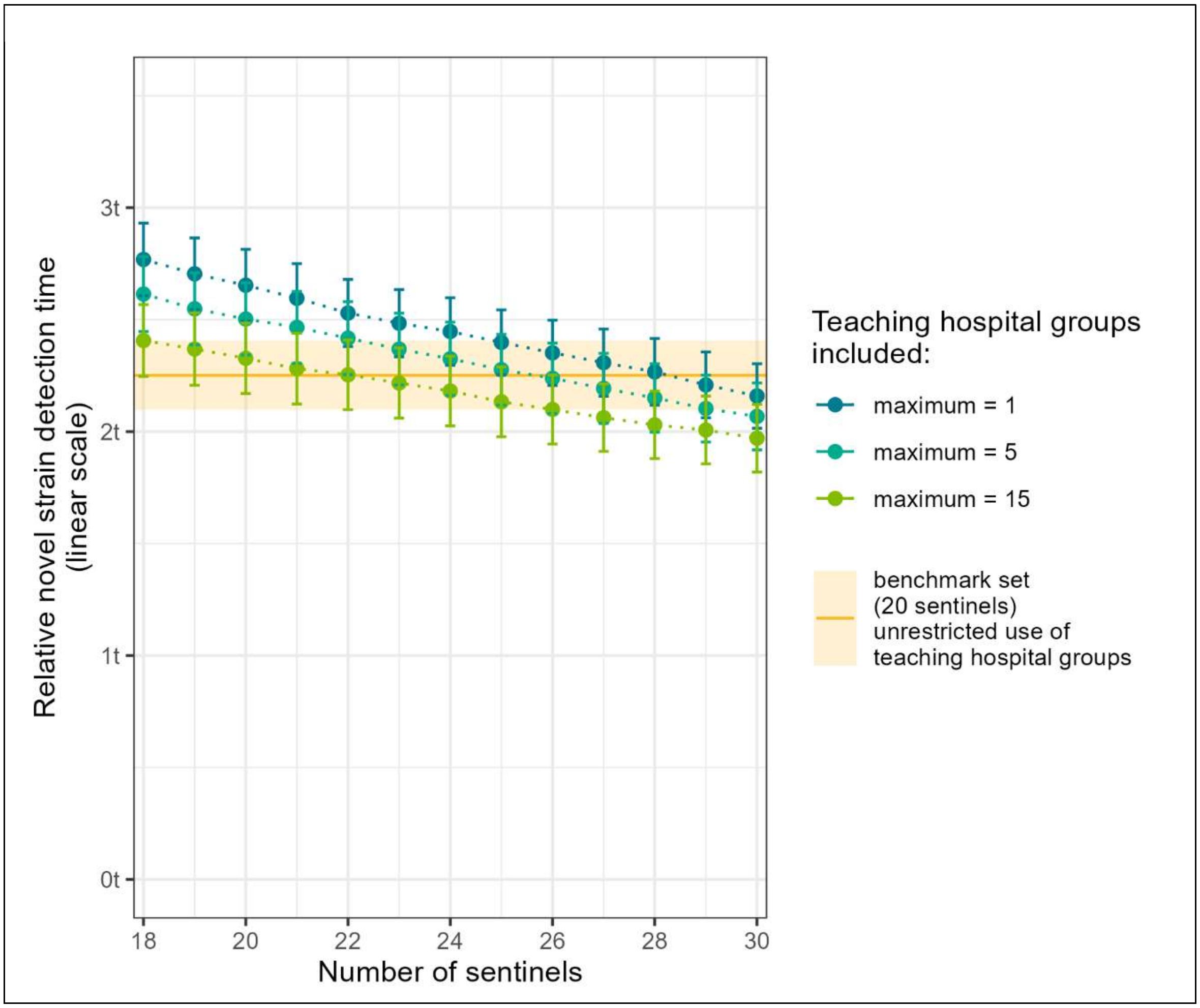

Supplementary Figure 2: Impact of restricting the number of teaching hospital groups on the mean time to detection vs unrestricted use by the benchmark set (S_0)

Detection time (mean +/-SD) by given size sentinel set taken from priority list obtained with a maximum of {15, 5, 1} teaching hospital groups used vs the benchmark 20-member sentinel set (S_0) which has no restriction on teaching type use.

Note: all results based on 13,700 simulations with same parameters (β_0, w=182 days) as for S_0; detection times are quoted relative to median detection time 't' using set S_0 and 10 specimens per month protocol

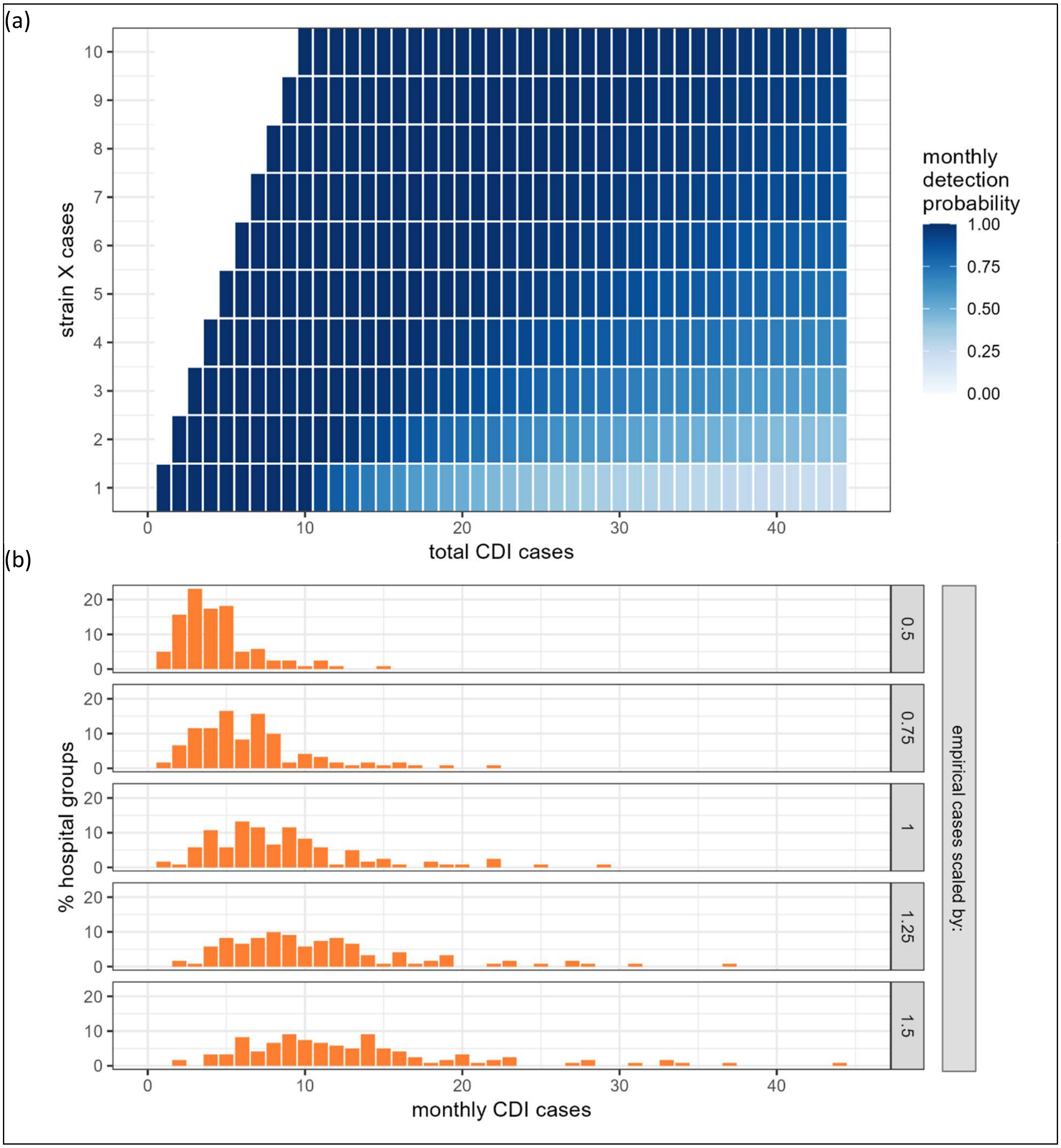

Supplementary Figure 3: Probability of detection within a month under protocol sampling 10 CDI cases per month (cf monthly CDI cases per hospital group)

a) Probability of detection within a month under protocol sampling 10 CDI cases per month: heat map by monthly CDI cases, strain X vs total. Values are generated by equation (3) in Methods section, with probability of detection = $1 - P_{monthly\ fail}$

b) Histogram of monthly CDI cases per hospital group for each CDI total incidence scenario: empirical monthly CDI cases scaled by {0.5, 0.75, 1, 1.25, 1.50}. Empirical CDI cases are mean values from three years of mandatory CDI surveillance[1] (FY2016/17-FY2018/19): source data are located in the source data file.

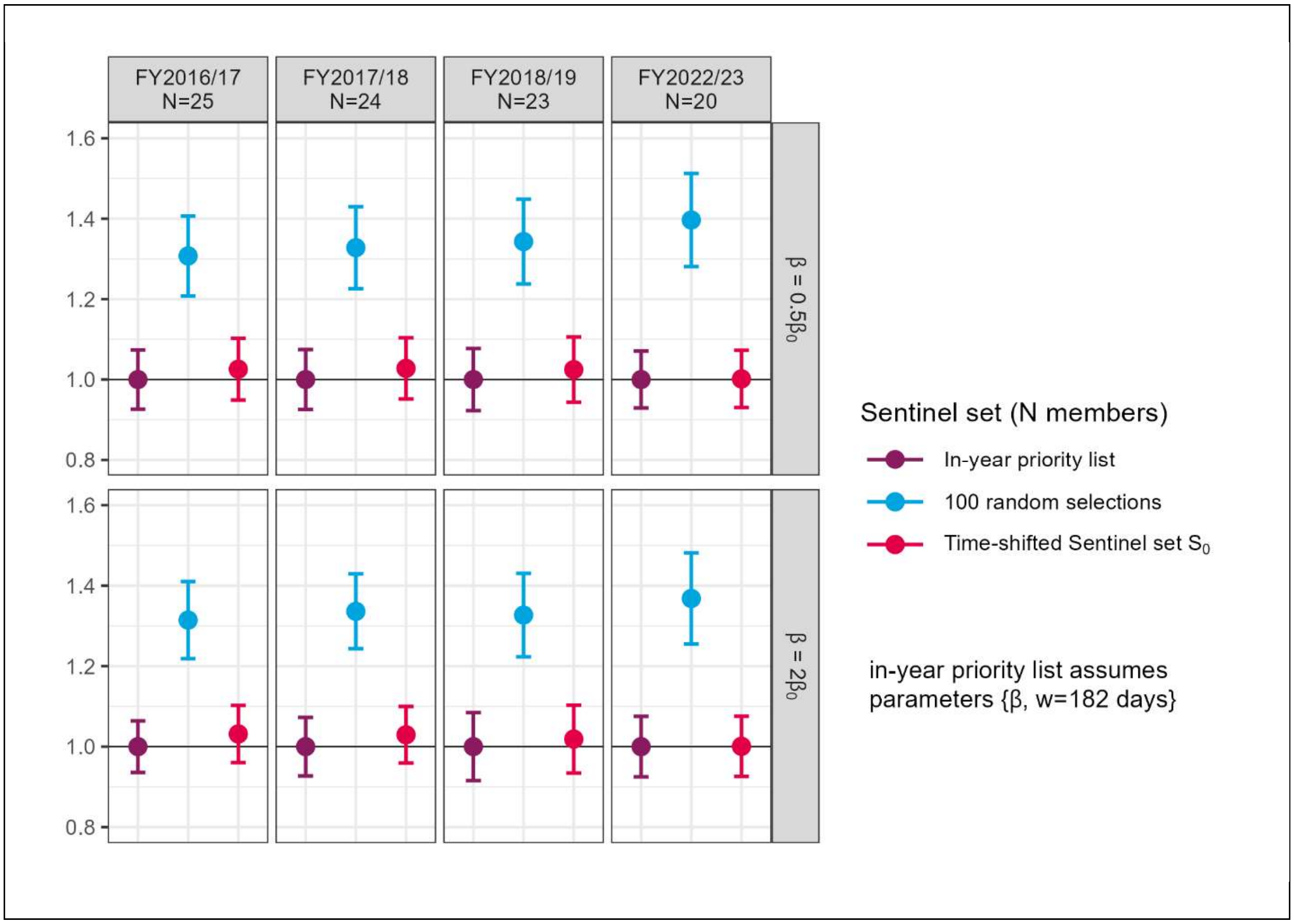

Supplementary Figure 4: In-the-past scenario analyses using transmission parameters ½β_0 and 2β_0

Under different assumptions for transmission parameter, β: detection times (mean ±SD) for S_0 (time-shifted from FY2022/23 to each year), in-year prioritised sentinel set and 100 randomly selected sets of N hospital groups, where N is the size of the time-shifted S_0.
Detection times in each scenario are shown as index vs detection time by corresponding in-year prioritised set; 13,700 simulations per scenario. Simulations and in-year prioritisation use specified β and w=182 days.

Reference for Supplementary Figures

1. United Kingdom Health Security Agency. *Clostridioides difficile* (*C difficile*) infection (CDI): annual data. (2024).
