# Supplementary material for: Novel surveillance design exploiting network analysis for efficient, rapid detection of emerging Clostridioides difficile strains
Source: Nat Commun. 2026 Aug 26;17:9051. doi: 10.1038/s41467-026-72311-0 (PMC13518965; doi:10.1038/s41467-026-72311-0)
Supplement: Supplementary file 2 [file 41467_2026_72311_MOESM2_ESM.pdf]

nature portfolio

Peer Review File

Novel surveillance design exploiting network analysis for efficient, rapid detection of emerging *Clostridioides difficile* strains

Corresponding Author: Dr Diane Pople

This file contains all reviewer reports in order by version, followed by all author rebuttals in order by version.

Version 0:

Reviewer comments:

Reviewer #1

(Remarks to the Author)
This is an impressive study performed by a very highly skilled team of researchers.
Using network analyses and empirical data from England they demonstrate the potential to select sentinel sites and hence reducing the number of C diff isolates needed for sequencing to quickly detect transmission between hospitals and outbreaks. The results were robust in several sensitivity analyses.

Overall, the analyses are well performed, the manuscript is clearly presented, and the conclusions are sound.

I have a couple of points/questions that should be considered to improve the manuscript.

1. The analyses are based on hospital groups not individual hospitals. Howe does this affect the internal and external validity of the results? Why not use individual hospitals rather than groups? I assume different groups contain different number of hospital and not all hospitals in a group will have the same amount of transfer of patients. How is this dealt with in the analysis? What are the assumptions that must be meet within hospital groups? What are the theoretical and practical implications of using hospital groups?
2. The authors account for average national prevalence of C. difficile carriage. How was local hospital carriage prevalence accounted for?
3. What is the evidence to support the assumption of carriage and transfer of C diff during 182 days (although the authors vary this by a shorter inter-stay window of 91 days)? How sensitive are the model results from varying carriage length between individuals?
4. Would it be possible to model a contact tracing scenario? I.e. when a new C diff variant is detected to perform sequencing in hospitals where there is lots of transfer from that hospital. Could this be a quicker way of detected and curbing transmission of new C diff variants.
5. I believe it would be interesting to complement the analyses with a cost-effectiveness assessment.
6. Figure 4a. Y axis is a bit difficult to understand. E.g. 2t from what? Is it possible to complement with an absolute time scale?

(Remarks on code availability)

Reviewer #2

(Remarks to the Author)
The manuscript is an impressive attempt to use network-based analysis and simulation modeling to identify how best to deploy WGS for CDI surveillance while avoiding the logistical burden of universal case testing. Overall, I found the manuscript clear, the results well reasoned, etc.

A few minor comments:

- Why the 182 day (i.e. six month) cut off? I suspect this is defensible, but does it come from a surveillance guideline, disease natural history, convenience, etc.?

- I would like to see some discussion as to whether the assumption that a hospital group is homogeneous (along with perhaps a notion for non-UK readers as to just how many facilities a hospital group might entail) is a reasonable assumption to make. It is certainly a *common* one made at both the group and facility level when looking at inter-facility transfer networks, but if there is data to show this is justified by more than computational efficiency, it would be nice to see that.

- The first panel of Figure 3b is somewhat poorly explained.

(Remarks on code availability)
Code has provided dummy data for replication, and an understandable lack of "real" data, with guides for who and how to contact for those materials. The README seems sufficient as well.

Reviewer #3

(Remarks to the Author)
This manuscript, entitled “Review: Exploiting network analysis to create a novel sentinel surveillance system for efficient, rapid detection of emerging Clostridioides difficile strains in England” by Diane Pople et al., presents a network of shared patients using national admission data to develop a new surveillance system for C. difficile. I was invited to provide expert feedback on the network analyses conducted in this study.

I would like to congratulate the authors on this excellent and innovative work. However, I have a few comments that may be worth considering:
First, could the authors elaborate on the in-hospital policies for C. difficile detection and screening during the study period? Have these policies changed over time, and if so, is this variation accounted for in the model? I understand that this is a model with hypothetical data for a new C.dif strain, however, increased screening could lead to higher detection rates, which may influence the network structure and should therefore be considered while modelling. If hospital-level data are unavailable, perhaps national screening guidelines could be referenced to contextualize the findings.
Second, it would be helpful to clarify the rationale behind the chosen definitions for the pandemic and post-pandemic periods. The World Health Organization officially declared the end of the COVID-19 pandemic in May 2023.
The current model includes limited post-pandemic data. While the limitations section mentions that robustness will be reassessed as more data become available, it might be useful to elaborate further on how this limitation could affect the current findings. Additionally, highlighting the specific time periods used in the analysis more clearly in the results section would help readers better understand the temporal scope and context of the conclusions.

(Remarks on code availability)

Version 1:

Reviewer comments:

Reviewer #4

(Remarks to the Author)
I was asked only to assess the authors' responses to original reviewer #2's comments, as that reviewer was not available to review the revised manuscript.

The original reviewer had only minor suggestions for clarifications and additional discussion, which the authors have addressed more than adequately in my opinion. The assumptions of the "182 day window" and of hospital group homogeneity were each well explained and justfied with added text and citations, and Figure 3 and its legend were modified substantially to improve and clarify its description of the depicted results.

(Remarks on code availability)

Open Access This Peer Review File is licensed under a Creative Commons Attribution 4.0 International License, which permits use, sharing, adaptation, distribution and reproduction in any medium or format, as long as you give appropriate credit to the original author(s) and the source, provide a link to the Creative Commons license, and indicate if changes were made.
In cases where reviewers are anonymous, credit should be given to 'Anonymous Referee' and the source.
The images or other third party material in this Peer Review File are included in the article's Creative Commons license, unless indicated otherwise in a credit line to the material. If material is not included in the article's Creative Commons license and your intended use is not permitted by statutory regulation or exceeds the permitted use, you will need to obtain permission directly from the copyright holder.
To view a copy of this license, visit https://creativecommons.org/licenses/by/4.0/

RESPONSE TO REVIEWER 1

Reviewer #1 (Remarks to the Author)

This is an impressive study performed by a very highly skilled team of researchers. Using network analyses and empirical data from England they demonstrate the potential to select sentinel sites and hence reducing the number of C diff isolates needed for sequencing to quickly detect transmission between hospitals and outbreaks. The results were robust in several sensitivity analyses.

Overall, the analyses are well performed, the manuscript is clearly presented, and the conclusions are sound.

I have a couple of points/questions that should be considered to improve the manuscript.

1. The analyses are based on hospital groups not individual hospitals. Howe does this affect the internal and external validity of the results? Why not use individual hospitals rather than groups? I assume different groups contain different number of hospital and not all hospitals in a group will have the same amount of transfer of patients. How is this dealt with in the analysis? What are the assumptions that must be meet within hospital groups? What are the theoretical and practical implications of using hospital groups?

The decision to use these hospital groups (known within NHS England as "acute hospital trusts") as sentinel sites, and hence their use in this analysis, was driven by their role as fundamental units for hospital care in England. These organisations, not individual hospitals, are the contracted operational providers for NHS secondary/tertiary care[1] and they act as reporting units, with many statistics only available at "trust" granularity. Specific to the context of *C. difficile*, in the mandatory CDI surveillance every CDI case is assigned to a "trust" but not necessarily to an individual hospital[2] and "trusts" are the entities contracted by NHSE for the control of CDI[3].

Therefore using these hospital groups as sentinels has the practical benefit for the WGS surveillance that, should a novel strain case be detected, it facilitates feedback to an operational organisation with responsibility for local interventions. It also has a modelling benefit, as the values of total CDI cases (used in the sampling protocol analysis and sampling burden estimation) are directly informed by empirical data[7]. We have expanded our description of the network vertices, to clarify these organisations' roles within the NHS and as source of comprehensive CDI case data:

> "Network vertices (or nodes) were acute hospital trusts: secondary/tertiary care-provider organisations within NHS England who are responsible for the control and reporting of CDI cases[30,31] subsequently denoted 'hospital groups'."

The reviewer is correct to surmise that these "trusts" can include more than one physical hospital site, however NHS data show that the majority contain either only one (acute) hospital or have a single dominant large site[4] which is responsible for most patient movements[5]. Hence homogeneity within hospital group is a model assumption, but reasonable in the above context (i.e. modal hospital group composition and granularity of CDI mandatory surveillance) and has been used in previous network analyses of England acute hospitals[5,6]. We have amended our original statement referring reader to Ciccolini *et al*'s discussion of hospital groups[5] to explicitly include details of modal hospital group composition and homogeneity assumption:

> "For clarity we repeat their observation[24] that a hospital group may legitimately be a multi hospital organisation in which case *C. difficile* presence might vary across component hospitals,

> just as within a single hospital it can vary across wards. However, in England, groups without a sole or dominantly large acute hospital form the minority[39]. Additionally these hospital groups, the institutions responsible for acute care in NHS England, are also the units for CDI mandatory reporting and accountability[30,31]; hence using them as the surveillance unit is a natural extension, and assumption of within group homogeneity is a reasonable model simplification."

2. The authors account for average national prevalence of C. difficile carriage. How was local hospital carriage prevalence accounted for?

This choice is imposed on us by the lack of granular data, which is a consequence of protocols regarding *C. difficile* testing. The national guidelines for *C. difficile* detection in place across the network analysis period[8] recommended the testing only of patients with symptoms of CDI (diarrhoea, as defined on the Bristol stool chart, which are is not otherwise clinically explained). Additionally, mandatory reporting protocol specifies exclusion of any instances of *C. difficile* detection in specimens taken from asymptomatic patients[2]. We have therefore clarified our rationale for using a national estimate within the online methods:

> "In the base analysis, we then adjusted each edge-weight (the number of patient movements) by estimated prevalence of *C. difficile* carriage (using a national estimate, 3%[33], in the absence of more granular data due to the symptomatic criteria required for testing[34] and reporting[30]) to scale to numbers of possible novel-strain-carrying vectors …"

Although we have been unable to include variation in carriage prevalence in patients discharged from different hospital groups within our modelling of strain X spread in our base analysis, due to lack of empirical data, we draw the reviewer's attention to the sensitivity analysis we conducted using alternative networks with edge-weights determined from the movements of patients with a known history of CDI in that period. This network definition results in variation by hospital group in the proportion of all patient movements made by potential vectors (median = 0.5%, with IQR 0.4%-0.7%, n = 137; weights summed across outgoing edges from each hospital group, comparing networks with w = 182 days). As reported in the manuscript (Results section headed "Sentinel set evaluation: sensitivity to novel strain assumptions"), we found the sentinel sets identified using these networks in the transmission modelling were acceptably stable vs those from the base analysis, with 15 sites present in all 20-member optimised sets obtained across a range of sensitivity analyses varying the inter-stay window, w, and transmission parameter, β, values.

3. What is the evidence to support the assumption of carriage and transfer of C diff during 182 days (although the authors vary this by a shorter inter-stay window of 91 days)? How sensitive are the model results from varying carriage length between individuals?

Considering the trajectory of our potential vector patients, we chose this value informed by studies of carriage resulting from known nosocomial transmission and of maintenance of carriage whilst in the community. As referenced by Crobach *et al*'s review of *C. difficile* colonisation[9], there has been limited research on carriage duration and results are subject to censoring – due to discharge (in healthcare-based studies), loss to follow-up and the study duration itself. Hospital-based studies indicate that over 80% of newly nosocomially-colonised asymptomatic patients are still positive at discharge (Samore et al 1994, McFarland et al 1989); a recent study[10] with a six-month follow-up period reported "persistent carriage" in 39% of such

patients (n=82) with median duration of carriage 77 days (range 7-133 days), however 95% confidence intervals are wide e.g. spanning approximately 0% - 60% at the last observation point. Community-based studies of healthy individuals report 17%-18% of participants remaining positive throughout the study duration (7 months in Kato et al 2001, 1 year in Ozaki et al 2004). We have added specific citations to the manuscript at the point where the 182 day value is introduced and have summarised sources in the online methods:

> "The maximum 182 day timespan between hospital stays was informed by studies of patients newly asymptomatically colonised with *C. difficile* whilst in hospital[9,32] and of *C. difficile* carriage/excretion duration within the community[9]."

There is a paucity of empirical data, and more studies to reduce the uncertainty are clearly needed, but findings to date suggest that 182 days is a reasonable limit to capture the admission of potential vectors whilst they are still capable of transmitting *C. difficile* to other patients. As noted by the reviewer, we also included the results of a sensitivity analysis with a 91 day window, in which we obtained a very similar sentinel selection to the base analysis.

We agree with the reviewer that we would expect variation of carriage duration by individuals, although this cannot be explicitly quantified in the above context of limited research in this area. However, the overall distribution of carriage duration (censored at 182 days by the movement data window) is implicitly included within the calibration of the transmission model to the real-world transmission of RT027, and thus is accounted for within the sentinel set benchmark selection. For clarity, we have added commentary on this implicit incorporation of carriage variation (despite the general paucity of explicit carriage data) to the online methods:

> "This approach implicitly incorporates the effect of the overall distribution of carriage duration (censored at the window length w), despite the lack of explicit granular data on carriage prevalence and duration."

4. Would it be possible to model a contact tracing scenario? I.e. when a new C diff variant is detected to perform sequencing in hospitals where there is lots of transfer from that hospital. Could this be a quicker way of detected and curbing transmission of new C diff variants.

We thank the reviewer for their suggestion to consider how the network analysis information compares with any inter-hospital epidemiological connections revealed through contact tracing from a case with the novel strain. In this analysis, though, we focus on the selection of sentinels using all patient movements to enable that first detection to happen promptly, reflecting the fact that patients can be vectors for transmission without having been identified as carriers. Hence the model is designed for the period before the existence of a novel strain becomes known.

We would expect that the response to any new strain detection would be thorough investigation, including contact tracing for that first known novel strain CDI case. At that future point the comparison suggested by the reviewer will become possible, and it will be a valuable and interesting analysis to undertake. As this aspect is rather different to the focus of the manuscript, we have not added further Discussion on the point, but could do so if the Editors preferred.

5. I believe it would be interesting to complement the analyses with a cost-effectiveness assessment.

RESPONSE TO REVIEWER 1

We agree that a complementary cost effectiveness analysis would be interesting to see, especially as cost is one of the factors that contributes to the capacity constraints for WGS.

Practical considerations, including budgetary, were central to the development of our approach seeking to harness the power of sequencing insight within limited capacity. However the precise components of a cost effectiveness analysis would need to reflect the specifics of any new strain e.g. increased transmission only, increased virulence only, or changes in transmission and virulence together. In contrast, both initial capital investment and ongoing implementation costs are directly related to sequencing effort. Hence, as part of implementation planning, the estimated sample burden was determined and it was considered by managers with financial responsibility in consultation with experts familiar with outbreak management costs. We have amended the manuscript to make explicit the link between sequencing burden and cost consideration:

> "For implementation, we required an estimated distribution of annual sequencing specimen burden, for comparison with capacity and to inform surveillance budget estimates."

Although a detailed cost effectiveness analysis is beyond the scope of the current model, we have clarified the inclusion of financial considerations within the manuscript's description of planned post-implementation service reviews:

> "Hence, once more post-pandemic data are available, robustness will be re-tested as part of post-implementation service evaluation, which will also consider a formal cost analysis (subject to appropriate empirical data)."

6. Figure 4a. Y axis is a bit difficult to understand. E.g. 2t from what? Is it possible to complement with an absolute time scale?

The y-axis in figure 4a shows the time from the emergence of strain X, i.e. when the index case facility becomes *AFFECTED*, until it is detected by the given sentinel set, i.e. a specimen from a patient with strain X CDI is included in sequencing sample of any of the sentinel sites. We have added this clarification to the Figure description and thank the reviewer for bringing this omission to our notice:

> "*… strain X detection time by a sentinel set is the time from the index case seeding until the earliest detection by any sentinel site(s) …"*

We use a time scale labelled with relative values to match the analysis's objective i.e. to minimise detection time (a relative measure). An absolute objective was rejected because time until detection would be strongly influenced by the rate of strain X spread, which depends on characteristics that are inherently unknown for a novel strain. In all the analyses, we report sentinel performance using relative comparisons within each parameter set, i.e. on a like-for-like basis, as absolute time could tempt cross-scenario comparisons which would be confounded by pathogen and network characteristics. The choice of the reference point "t" for graphical purposes is arbitrary, but a definition is given the notes to Figure 4.

RESPONSE TO REVIEWER 2

Reviewer #2 (Remarks to the Author)

The manuscript is an impressive attempt to use network-based analysis and simulation modeling to identify how best to deploy WGS for CDI surveillance while avoiding the logistical burden of universal case testing. Overall, I found the manuscript clear, the results well reasoned, etc.

A few minor comments:

- Why the 182 day (i.e. six month) cut off? I suspect this is defensible, but does it come from a surveillance guideline, disease natural history, convenience, etc.?

The 182 day window between hospital admissions (in the base analysis) was based on the natural history of *C. difficile* colonisation. We refer to our response to Reviewer 1 (question 3) including a summary of the relevant literature and amendments to the manuscript to clarify the parameterisation support.

We also draw the reviewer's attention to the sensitivity analysis which used an alternative 91 day window (reported in the Results section headed "Sentinel set evaluation: sensitivity to novel strain assumptions"), in which we obtained a very similar sentinel selection to the base analysis.

- I would like to see some discussion as to whether the assumption that a hospital group is homogeneous (along with perhaps a notion for non-UK readers as to just how many facilities a hospital group might entail) is a reasonable assumption to make. It is certainly a *common* one made at both the group and facility level when looking at inter-facility transfer networks, but if there is data to show this is justified by more than computational efficiency, it would be nice to see that.

We agree with the reviewer's observation that hospital group homogeneity is a common assumption when working with transfer networks; this assumption is, for example, present in the network analysis studies that we build upon in this work[5,6].

We refer to our response to Reviewer 1 (question 1) including the corresponding manuscript amendments which expand the network vertex definition and provide the modal hospital group composition, giving additional context for non-UK readers. These additional details clarify our motivation for using these hospital groups and provide support for our assumption of within group homogeneity.

Additionally we note that, beyond specifics for the exemplar of *C. difficile* in England, the methodology we describe is applicable to selection of individual hospitals as sentinels in circumstances where necessary supporting data are available and would be appropriate regarding implementational considerations.

- The first panel of Figure 3b is somewhat poorly explained.

Thank you for this feedback and the opportunity to improve the clarity with which this information is presented to readers. We have revised figure 3, splitting the information conveyed in the original double-plot Figure 3b into two panels (3b and 3c), which enables us to better explain each plot.

RESPONSE TO REVIEWER 2

The new panel 3b uses a more conventional left-to-right x-axis, and includes binned data for all delays (between a hospital group gaining Affected status and strain X detection) not just the proportion of zero-value delays. Panel 3c provides more detail on the distribution of non-zero-value delays. We have also added a reference to the supplementary material, which assists comprehension of the similar zero-value proportions in panel 3b (i.e. primarily due to the hospital groups which have no more than 10 cases per month across all strains, so the first strain X case is necessarily included in the specimens sequenced).

For the reviewer's convenience we include a copy of the new panels here and the legend, which we have also expanded to increase clarity (panel 3a unchanged except amending x-axis title to match the legend wording i.e. "time until all hospital groups Affected (years)"):

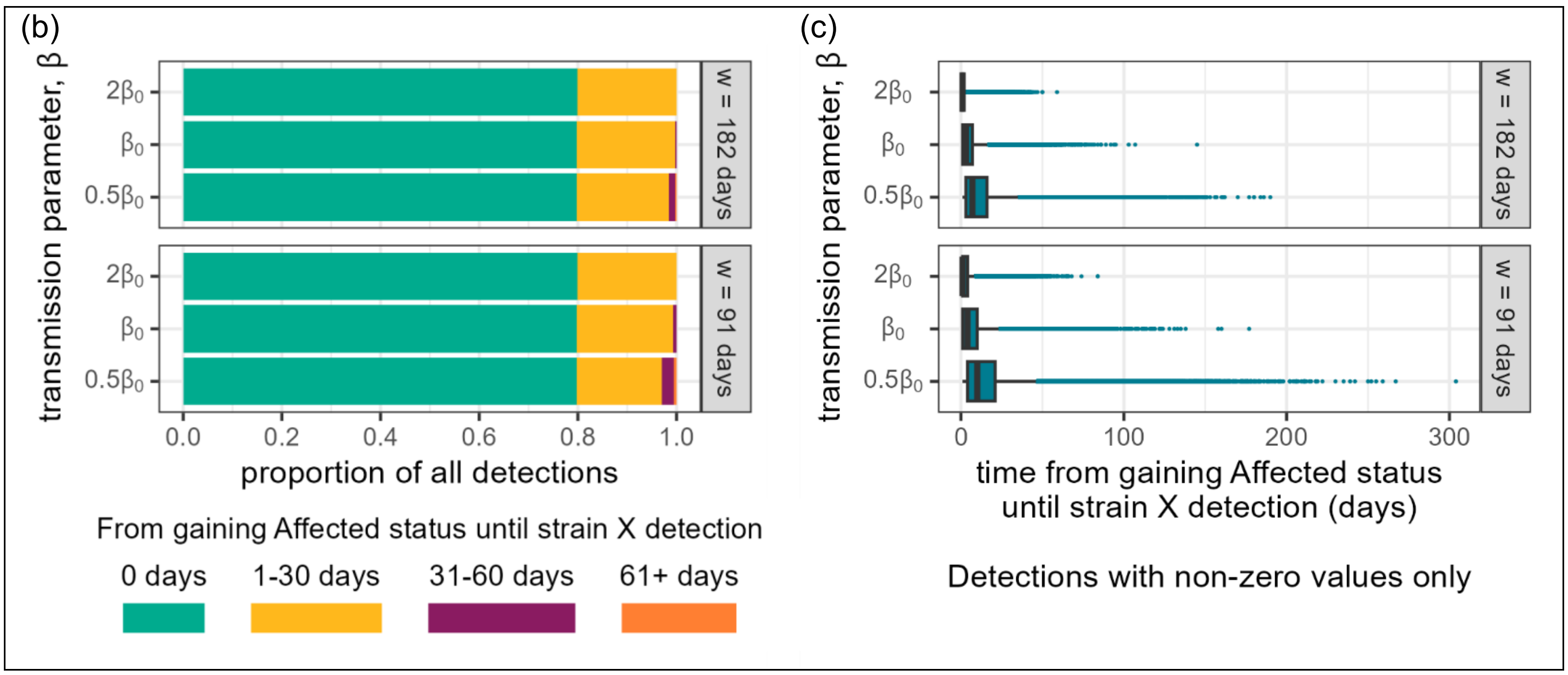

Figure 3: Spread and detection of a novel strain (strain 'X')

Under different assumptions (transmission parameter, β, and window between inpatient hospital stays, w):
(a) time (years) from index case seeding until all hospital groups have *AFFECTED* status (n=13,700 simulations each)
(b) time (days) after hospital group gained *AFFECTED* status until strain X detected there under the 10 specimens per month protocol, all hospital groups*: proportion of all detections with 0, 1-30, 31-60 and 61+ days delay (n=1,876,900 detections each).
(c) time (days) after hospital group gained *AFFECTED* status until strain X detected there under the 10 specimens per month protocol: distribution for non-zero values only (n=377,478 to n=379,407 detections each, see panel b)

*Hospital groups with ≤10 CDI cases per month always have no delay between a hospital group gaining *AFFECTED* status and strain X detection, by assumption, under this protocol (see Supp. Fig. 3b).

NB always no delay between a hospital group gaining *AFFECTED* status and strain X detection, by assumption, under the all-cases protocol.

Note: boxplots show interquartile range (IQR), whiskers (in black) at 1.5 IQR and outliers (in teal), 100 simulations per index case facility (each of the 137 acute hospital groups in FY2022/23 network), detections at each hospital group in each simulation. Censored data for incomplete process at 7,300 days (a) 10 simulations

RESPONSE TO REVIEWER 2

($0.5\beta_0$, w=182) and 555 simulations ($0.5\beta_0$, w=91), (b) 11 detections ($0.5\beta_0$, w=182) and 1280 detections ($0.5\beta_0$, w=91)

Reviewer #2 (Remarks on code availability)

Code has provided dummy data for replication, and an understandable lack of "real" data, with guides for who and how to contact for those materials. The README seems sufficient as well.

We thank the reviewer for their time looking at these elements of our submission and we are grateful for their positive feedback on them all. We are pleased that they understand the reasons why “real” data could not be shared and that they the recognise the steps we have taken to enable readers to still run example analyses given those circumstances or to seek access to the original data themselves.

RESPONSE TO REVIEWER 3

Reviewer #3 (Remarks to the Author)

This manuscript, entitled "Review: Exploiting network analysis to create a novel sentinel surveillance system for efficient, rapid detection of emerging Clostridioides difficile strains in England" by Diane Pople et al., presents a network of shared patients using national admission data to develop a new surveillance system for C. difficile. I was invited to provide expert feedback on the network analyses conducted in this study.

I would like to congratulate the authors on this excellent and innovative work.

However, I have a few comments that may be worth considering:

First, could the authors elaborate on the in-hospital policies for C. difficile detection and screening during the study period? Have these policies changed over time, and if so, is this variation accounted for in the model? I understand that this is a model with hypothetical data for a new C.dif strain, however, increased screening could lead to higher detection rates, which may influence the network structure and should therefore be considered while modelling. If hospital-level data are unavailable, perhaps national screening guidelines could be referenced to contextualize the findings.

The same national guidance to healthcare providers regarding *C. difficile* diagnosis[8] remained in place from 2012-2025, covering the whole period used for network generation (pre- and post-pandemic) and for the estimation of total number of CDI cases (in the sampling protocol analysis and the WGS sequencing sample burden). This guidance recommended the testing of all patients with symptoms of CDI (notably diarrhoea) which are not otherwise explained and a two-stage testing approach (a glutamate dehydrogenase (GDH) enzyme immunoassay (EIA) test followed by a sensitive toxin EIA test). *C. difficile* screening of asymptomatic patients is not recommended.

Individual hospital group policies are not published externally or otherwise centrally available. However the assumption of consistent adoption of these guidelines, both longitudinally and geographically, is reasonable given the long-term stability of the guidelines and that 94% of hospital groups reported compliance soon after their introduction[11], with similar findings in a more recent survey[12] (89% national guideline awareness and 95% appropriate assay use, n = 20 UK hospitals).

We have added guidelines citation to where we first refer to the CDI cases empirical data:

> "We assumed total CDI cases at each hospital group matched mean monthly CDI case[34] numbers from mandatory surveillance[35] …"

and clarified the testing recommendations which were applicable and widely adopted across the full analysis period (FY2016/17, FY2017/18, FY2018/19 and FY2022/23) within the online methods:

> "National guidance on CDI case diagnosis[34] was invariant across analysis period, with evidence of widespread local implementation[59,60]. This recommended testing of patients with CDI symptoms (diarrhoea) using a two-stage testing approach (a glutamate dehydrogenase (GDH) enzyme immunoassay (EIA) test followed by a sensitive toxin EIA test); screening of asymptomatic patients for *C. difficile* carriage was not recommended."

Whilst testing homogeneity is a reasonable assumption, we further note that although is it possible that local policy variation could affect detection, the main model does not rely on this, but rather uses all patient movements, assuming all individuals are potentially unsampled

vectors. We demonstrate its robustness through sensitivity analyses including comparison of the sentinel sites selected restricting potential vectors to those with known CDI (identified through whatever local testing policies happened to be in place). We have not amended the manuscript as we consider the Discussion already covered the robustness of our selection, but could add more details if the Editors preferred.

We note that there is evidence of greater variation in the tests used[13] during the earlier period used for transmission spread calibration (i.e. the emergence of RT027), although by 2011[11] 79% of hospital groups already reported policies which would be consistent with the later national guidance. We have added a caveat regarding testing variation during RT027 emergence to the discussion of calibration data limitations:

> "… we were unable to adjust for any confounding from differences between contemporary networks vs those in place when RT027 cases were rising and for the impact on transmission from temporal differences in infection prevention and control (IPC) practices or relating to variation in CDI testing vs current guidance."

The reviewer asks us to consider increased screening. We have already provided in the original submission evidence that current practice captures nearly all CDI cases[14], however we are unsure if the reviewer is suggesting that sentinel sites be encouraged to widen testing criteria which might permit detection of asymptomatic patient vectors (and highlighting that modelling consequences of any resultant patient-specific interventions triggered by a positive test would require consideration). This is not a direction we have explored because this analysis is focused determining optimal selection of sentinel sites to deliver "identification of novel *C. difficile* strains within real-world logistical constraints". The results of our analysis have enabled realisation of this objective without increasing the test burden on participating local laboratories, as well as within the available national laboratory WGS sequencing capacity. For clarification we have made explicit this benefit of our approach in the first paragraph of the discussion:

> "We have achieved this without additional patient sample collection; however, implementing a new surveillance system requires investment in people, resources and systems which may prompt real-world considerations beyond laboratory capacity …."

Second, it would be helpful to clarify the rationale behind the chosen definitions for the pandemic and post-pandemic periods. The World Health Organization officially declared the end of the COVID-19 pandemic in May 2023. The current model includes limited post-pandemic data. While the limitations section mentions that robustness will be reassessed as more data become available, it might be useful to elaborate further on how this limitation could affect the current findings.

We thank the reviewer for their close reading of the data period definitions in comparison with the global SARS CoV-2 pandemic timeline and have amended the manuscript to clarify our rationale. The pandemic exclusion period was based on the presence of government-mandated community-based control measures which would necessarily affect the movement of potential vectors. In England these were place at various times during FY2019/20 through FY2021/22[15,16]. We assume after this point patients were fully free to resume a 'normal' pattern of hospital attendance. We have clarified the post pandemic definition in the manuscript:

> "… complete data for analysis were available for one post pandemic year only (i.e. after legal movement restrictions were removed[50])."

RESPONSE TO REVIEWER 3

We have also included a post-pandemic dates rationale to the online methods:

> "This post-pandemic period was after the relaxation of the final government-mandated community-based measures which would necessarily affect the movement of potential vectors[50,55], we assume patients were thus able to resume a 'normal' pattern of hospital attendance."

As noted by the reviewer, we already have plans to revisit the analysis once more years of post-pandemic data become available. In the meantime, the temporal generalisability scenario analysis (using three different pre-pandemic periods) demonstrated the benchmark set's robustness – comparing both the optimum sentinel set composition and detection time performance vs contemporaneous data analysis. We also considered each dataset's susceptibility to short-term fluctuations, for example we minimised the impact of local outbreaks by employing multi-year averages for the hospital group CDI case data where possible. By contrast the benchmark analysis used all patient movements in determining the network, data which are less vulnerable to these short-term localised effects (although not the systemic shock of the SARS-CoV-2 pandemic and associated legislation: hence this period's exclusion from the analysis). We have expanded the previous discussion of the temporal analysis to make explicit these considerations:

> This is mitigated by the robust performance of the benchmark sentinel set when applied retrospectively, for both composition and performance metrics. CDI case data at hospital group granularity are vulnerable to temporal fluctuation from local outbreaks; hence multi-year averages were used in preference to single-year data (in modelling sampling protocols and sample burden estimation). This was not possible with the linked CDI case movement data, but these were only used in one sensitivity analysis, limiting potential influence on the overall results.

Additionally, highlighting the specific time periods used in the analysis more clearly in the results section would help readers better understand the temporal scope and context of the conclusions.

We define all four Financial Year periods included in the analyses within the results section headed "Empirical network identification and characteristics". We appreciate the reviewer bringing to our attention that this is not close to where the temporal stress-testing results are presented; hence we have added specific dates for all periods as notes to the corresponding figure (Figure 6) to reduce the need for readers to refer back to the earlier text.

References in responses to Reviewers

1. NHS England. NHS Data Model and Dictionary : Health Care Provider Business Definition. (2025).
2. United Kingdom Health Security Agency. Mandatory enhanced MRSA, MSSA and Gram-negative bacteraemia, and Clostridioides difficile infection surveillance. Protocol 4-4. (2021).
3. National Health Service England. Minimising Clostridioides difficile and Gram-negative Bloodstream Infections. (2021).
4. NHS Digital. Estates Returns Information Collection. (NHS England, 2024).
5. Ciccolini, M., Donker, T., Grundmann, H., Bonten, M.J. & Woolhouse, M.E. Efficient surveillance for healthcare-associated infections spreading between hospitals. *Proc Natl Acad Sci U S A* **111**, 2271-2276 (2014).
6. Donker, T.*, et al.* Using hospital network-based surveillance for antimicrobial resistance as a more robust alternative to self-reporting. *PLoS One* **14**, e0219994 (2019).
7. United Kingdom Health Security Agency. Clostridioides difficile (C difficile) infection (CDI): annual data. (2024).
8. Department of Health. Clostridium difficile: updated guidance on diagnosis and reporting. (2012).
9. Crobach, M.J.T.*, et al.* Understanding Clostridium difficile Colonization. *Clin Microbiol Rev* **31**(2018).
10. Curry, S.R.*, et al.* Natural History of Clostridioides difficile Colonization and Infection Following New Acquisition of Carriage in Healthcare Settings: A Prospective Cohort Study. *Clin Infect Dis* **77**, 77-83 (2023).
11. Davies, K.A.*, et al.* Underdiagnosis of Clostridium difficile across Europe: the European, multicentre, prospective, biannual, point-prevalence study of Clostridium difficile infection in hospitalised patients with diarrhoea (EUCLID). *Lancet Infect Dis* **14**, 1208-1219 (2014).
12. Viprey, V.F.*, et al.* European survey on the current surveillance practices, management guidelines, treatment pathways and heterogeneity of testing of Clostridioides difficile, 2018-2019: results from The Combatting Bacterial Resistance in Europe CDI (COMBACTE-CDI). *J Hosp Infect* **131**, 213-220 (2023).
13. Goldenberg, S.D. & French, G.L. Diagnostic testing for Clostridium difficile: a comprehensive survey of laboratories in England. *J Hosp Infect* **79**, 4-7 (2011).
14. Agnew, E.*, et al.* Impact of testing on Clostridioides difficile infection in hospitals across Europe: a mathematical model. *Clin Microbiol Infect* **29**, 796.e791-796.e796 (2023).
15. United Kingdom of Great Britain and Northern Ireland. Coronavirus Act 2020 (c.7). (The Stationary Office, 2020).
16. Cabinet Office. COVID-19 Response: Living with COVID-19. (2022).

Individual carriage studies cited via review by Crobach et al

Samore, M.H.*, et al.* Clostridium difficile colonization and diarrhea at a tertiary care hospital. *Clin Infect Dis* **18**, 181-187 (1994).

McFarland, L.V., Mulligan, M.E., Kwok, R.Y. & Stamm, W.E. Nosocomial acquisition of Clostridium difficile infection. *N Engl J Med* **320**, 204-210 (1989).

Kato, H.*, et al.* Colonisation and transmission of Clostridium difficile in healthy individuals examined by PCR ribotyping and pulsed-field gel electrophoresis. *J Med Microbiol* **50**, 720-727 (2001).

Ozaki, E.*, et al.* Clostridium difficile colonization in healthy adults: transient colonization and correlation with enterococcal colonization. *J Med Microbiol* **53**, 167-172 (2004).
